# KAT2A/KAT2B-targeted acetylome reveals a role for PLK4 acetylation in preventing centrosome amplification

**DOI:** 10.1038/ncomms13227

**Published:** 2016-10-31

**Authors:** Marjorie Fournier, Meritxell Orpinell, Cédric Grauffel, Elisabeth Scheer, Jean-Marie Garnier, Tao Ye, Virginie Chavant, Mathilde Joint, Fumiko Esashi, Annick Dejaegere, Pierre Gönczy, László Tora

**Affiliations:** 1 Institut de Génétique et de Biologie Moléculaire et Cellulaire (IGBMC), 67404 Illkirch, France; 2 Centre National de la Recherche Scientifique, UMR7104, 67404 Illkirch, France; 3 Institut National de la Santé et de la Recherche Médicale, U964, 67404 Illkirch, France; 4 Université de Strasbourg, 67404 Illkirch, France; 5 Sir William Dunn School of Pathology, University of Oxford, South Parks Road, Oxford OX1 3RE, UK; 6 Swiss Institute for Experimental Cancer Research (ISREC), School of Life Sciences, Swiss Federal Institute of Technology Lausanne (EPFL), CH-1015 Lausanne, Switzerland; 8

## Abstract

Lysine acetylation is a widespread post-translational modification regulating various biological processes. To characterize cellular functions of the human lysine acetyltransferases KAT2A (GCN5) and KAT2B (PCAF), we determined their acetylome by shotgun proteomics. One of the newly identified KAT2A/2B substrate is polo-like kinase 4 (PLK4), a key regulator of centrosome duplication. We demonstrate that KAT2A/2B acetylate the PLK4 kinase domain on residues K45 and K46. Molecular dynamics modelling suggests that K45/K46 acetylation impairs kinase activity by shifting the kinase to an inactive conformation. Accordingly, PLK4 activity is reduced upon *in vitro* acetylation of its kinase domain. Moreover, the overexpression of the PLK4 K45R/K46R mutant in cells does not lead to centrosome overamplification, as observed with wild-type PLK4. We also find that impairing KAT2A/2B-acetyltransferase activity results in diminished phosphorylation of PLK4 and in excess centrosome numbers in cells. Overall, our study identifies the global human KAT2A/2B acetylome and uncovers that KAT2A/2B acetylation of PLK4 prevents centrosome amplification.

## Abstract (web summary)

The acetyltransferases KAT2A and KAT2B are essential regulators of transcription, cell cycle progression and DNA repair. Here the authors describe a KAT2A/2B-dependent acetylome, and show that acetylation of the protein kinase PLK4 contributes to the regulation of centrosome number.

Lysine (K) acetylation has emerged as a widespread post-translational modification that is conserved from prokaryotes to eukaryotes, and which regulates various biological processes^1^,^2^,^3^,^4^,^5^,^6^. Lysine acetylation can modify the charge of a given protein and/or create docking sites for other proteins that may alter their function^7^. A prime example of the significance of such modification is the acetylation of histones, which plays an essential role in transcriptional activation, DNA replication and repair^8^. Remarkably, in addition, lysine acetylation also targets thousands of non-histone proteins^1^,^2^, but the functional relevance of the vast majority of these modifications is not known.

Lysine acetylation is catalysed by K acetyltransferases (KATs; formerly histone acetyltransferases, HATs), which transfer the acetyl group of acetyl-CoA to the epsilon-amino group of internal lysine residues^9^. Whereas ∼6,000 proteins have been reported to be acetylated in human cells (Phosphositeplus^10^), only ∼20 KATs have been identified to date (reviewed in ref. 11), suggesting that each KAT could acetylate several hundred targets. Thus, it is important to identify the specific subset of proteins acetylated by each individual KAT. The human KAT2A (GCN5) and its ∼70% identical paralogue KAT2B (PCAF) are known to play a role in diverse biological processes, such as chromatin remodelling, transcriptional regulation, DNA replication, DNA repair, cell cycle progression and cell death^12^,^13^,^14^,^15^,^16^,^17^,^18^. KAT2A/2B are mainly studied as HATs that acetylate preferentially histone H3 and to a lesser extent H4 (ref. 19 and references therein), leading to changes in chromatin structure. However, KAT2A/2B can also acetylate non-histone targets, such as CDC6 and cyclin A to regulate the G1/S cell cycle transition and mitosis^13^,^14^. While KAT2A/2B have been implicated in given cellular processes, a comprehensive list of their cellular targets has not yet been assembled, although the identification of such targets should provide more mechanistic insights into their mode of action.

Metazoan KAT2A/2B function within several multiprotein coactivator complexes, such as SAGA (Spt-Ada-GCN5 acetyltranferase (AT) containing) and ATAC (Ada-Two-A-containing complex)^20^,^21^,^22^. We have previously shown that ATAC controls mitotic progression by acetylating cyclin A, and that impairing the activity of KAT2A/2B leads to increased centrosome numbers in mammalian cells^14^. However, the mechanisms underlying the requirement of KAT2A/2B in regulating centrosome numbers remained elusive.

Centrosomes consist of a pair centrioles surrounded by pericentriolar material, from which microtubules are nucleated in animal cells. Proliferating cells are typically born with two centrioles, which duplicate once per cell cycle, starting towards the G1/S transition. As a result, cells in S phase and thereafter contain two pairs of centrioles, each within one centrosome. At the G2/M transition, the two centrosomes separate to direct bipolar spindle assembly during mitosis. Aberrant centrosome number has dire consequences for cell division and genome integrity, since too few centrosomes can lead to monopolar spindle assembly and too many centrosomes to multipolar spindle assembly^23^,^24^,^25^. Centrosome amplification is frequently observed in human cancer and has been proposed to contribute to tumour progression^23^,^24^,^25^. Therefore, the precise regulation of the number of centrosomes is fundamental for human health.

In metazoans, a key regulator of centrosome number is the serine/threonine polo-like kinase 4 (PLK4)^26^,^27^. PLK4 depletion results in failure of centriole formation, whereas its overexpression leads to supernumerary centrioles^26^,^27^. Therefore, PLK4 protein levels and kinase activity must be tightly regulated. This is achieved in part by PLK4 protein stability being regulated by auto-phosphorylation, which triggers ubiquitin-mediated proteasomal degradation (reviewed in refs 28, 29, 30). Whereas the mechanisms regulating PLK4 activation, protein ubiquitination and degradation have been clarified, those modulating PLK4 kinase activity remain elusive.

In this study, we have determined the KAT2A/2B-dependent acetylome of human cells and identify 398 acetylated KAT2A/2B target proteins involved in diverse cellular processes. Furthermore, our detailed analysis uncovers that KAT2A/2B-mediated lysine acetylation of PLK4 negatively regulates its kinase activity and thus keeps in check the number of centrosomes in human cells, thereby contributing to the maintenance of genome integrity.

## Results

### Characterization of the human KAT2A/2B-dependent acetylome

**Figure 1:**
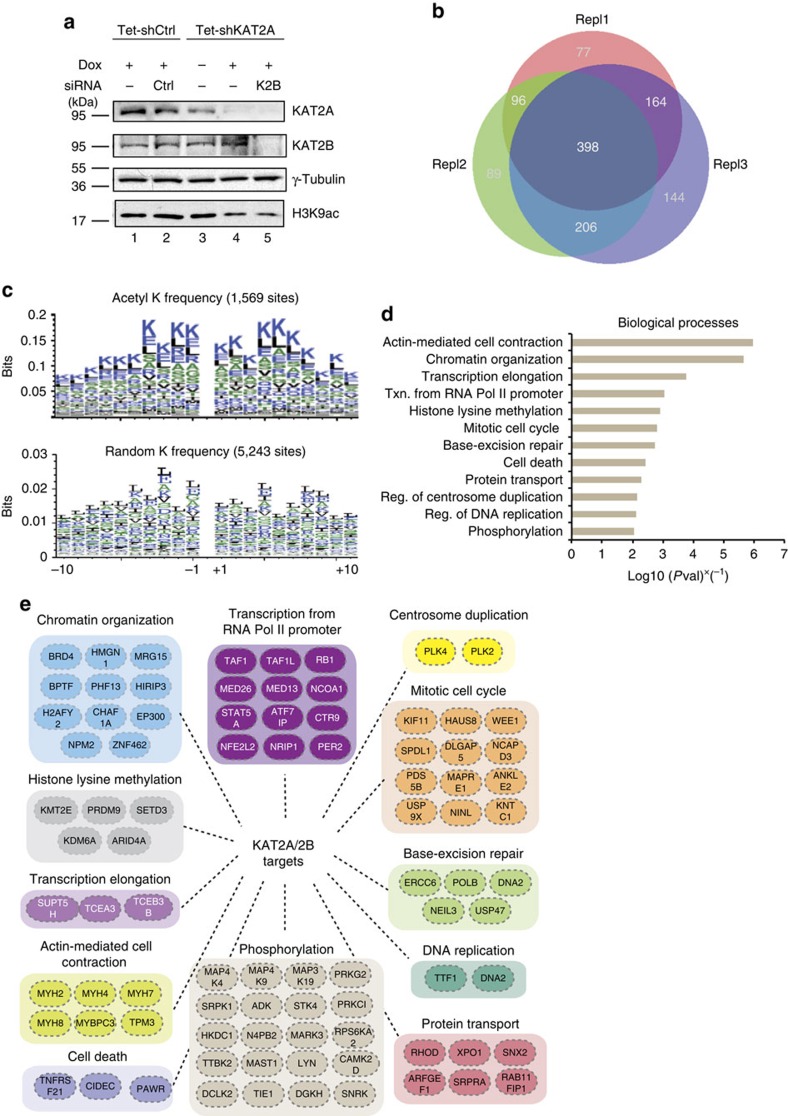
Identification of the KAT2A- and KAT2B-dependent acetylome. (**a**) KAT2A/2B knockdown efficiency. Tetracycline (Tet) inducible stable HeLa cell lines were used in which shRNAs either not targeting any endogenous transcript (Tet-shCtrl) or targeting KAT2A (Tet-shKAT2A) were expressed under doxycycline (Dox) induction. In the Tet-shKAT2A cell line, a siRNA against *KAT2B* (K2B) was also transfected. The knockdown efficiency of KAT2A, KAT2B and the acetylation of histone H3K9 was tested by western blot analyses. (**b**) In all, 398 potential KAT2A/2B acetylated protein targets were identified as being present in control samples (3/5), but absent in all KAT2A/2B KD-depleted cell lysates. (**c**) Analysis of the frequency of amino acids surrounding the acetylated lysines targeted by KAT2A and KAT2B, as compared with that of amino acids surrounding non-acetylated lysines. In all, 1,569 distinct ‘Ks' from the list of 398 proteins were used in this analysis, with 10 aa upstream (−10 on the *x* axis) and 10 aa downstream (+10 on the *x* axis). The K itself (at position 0) is not shown in these logos. A random selection of 5,243 sequences (K in the middle, 21 aa length, 0.05% of the total K) from the total human proteins was used for the comparison. The overall height of the stack indicates the sequence conservation at that position, while the height of symbols within the stack indicates the relative frequency of each amino acid at that position. Note that the *y* axes on the upper and lower graphs are different. (**d**) Gene ontology (GO) term enrichment analysis for biological processes using Manteia^34^ of the 398 identified proteins. Pathways at a false discovery rate below 1% are represented. The *x* axis represents the reverse *P* value obtained after GO term enrichment analysis in Manteia^34^. Txn,transcription. (**e**) Examples of proteins acetylated by KAT2A/2B belonging to the same biological pathways targeted by KAT2A/2B (see GO term analysis in **d**).

To comprehensively identify proteins acetylated specifically by endogenous KAT2A/2B (GCN5/PCAF), we performed a large-scale unbiased screen using tandem mass spectrometry (MS)-based shotgun proteomics, comparing acetylated proteins in control cells and in cells in which KAT2A and KAT2B were simultaneously knocked down. We used two stable HeLa cell lines in which doxycycline (Dox) induces the expression of either a control short-hairpin (sh) RNA (Tet-shCTRL) or a shRNA targeting KAT2A (Tet-shKAT2A)^14^. In addition, KAT2B was knocked down in the latter cell line by siRNA transfection (Fig. 1a). The efficiency of KAT2A and KAT2B knockdown was verified by western blot analysis (Fig. 1a) and further ascertained by the decrease of histone H3 acetylation at position K9, a well-known *in vivo* target of these KATs^31^ (Fig. 1a, lowest panel). The redundant acetylation of H3K9 by other nuclear KATs following the depletion of KAT2A/2B likely explains the remaining signal in lane 5 of Fig. 1a.

The acetylated peptide pool was then identified either under control conditions in five biological replicates or in the double KAT2A/2B knockdown cells in three biological replicates, and compared. Acetylated peptides and their corresponding proteins that were detected solely in the control cells (in a minimum of 3/5 replicates), but not in the knockdown conditions, were considered as potential targets of KAT2A/2B. This stringent cutoff led to the identification of 1,569 acetylated sites on 398 proteins (Fig. 1b and Supplementary Data 1). Among these KAT2A/2B targets, 251 proteins (about 63%) were already found to be acetylated in global acetylome screens (see the protein modification resource: PhosphositePlus^10^, Supplementary Fig. 1), validating our approach. The analysis of amino-acid frequency surrounding the identified acetylated lysines revealed enrichment for further lysine residues, as compared with the frequency of residues surrounding non-acetylated lysines (Fig. 1c and Supplementary Data 1). This result suggests that the amino-acid composition surrounding lysines influences the substrate specificity of KAT2A/B, which preferentially acetylate lysine-rich regions of proteins. This is in good agreement with previous reports showing an enrichment of lysines residues surrounding acetylated lysines in global human acetylome data sets^1^,^32^,^33^.

Gene ontology term analyses of the 398 non-histone proteins identified here using Manteia^34^ indicated that the KAT2A/2B acetylated proteins play a role in cellular functions in which KAT2A/2B have already been implicated, including chromatin remodelling, transcriptional regulation, DNA replication, DNA repair, cell death and cell cycle progression (Fig. 1d,e and Supplementary Data 1). Interestingly, the gene ontology term analysis also suggested KAT2A/2B-dependent regulation of novel pathways, such as actin-mediated cell contraction, protein transport, phosphorylation and regulation of centrosome duplication (Fig. 1d,e and Supplementary Data 1). Overall, these findings indicate that the action of KAT2A/2B is more widespread than previously suspected and uncover novel processes by which these KAT2s might modulate cell physiology.

### The kinase domain of PLK4 is acetylated by KAT2A and KAT2B

**Figure 2:**
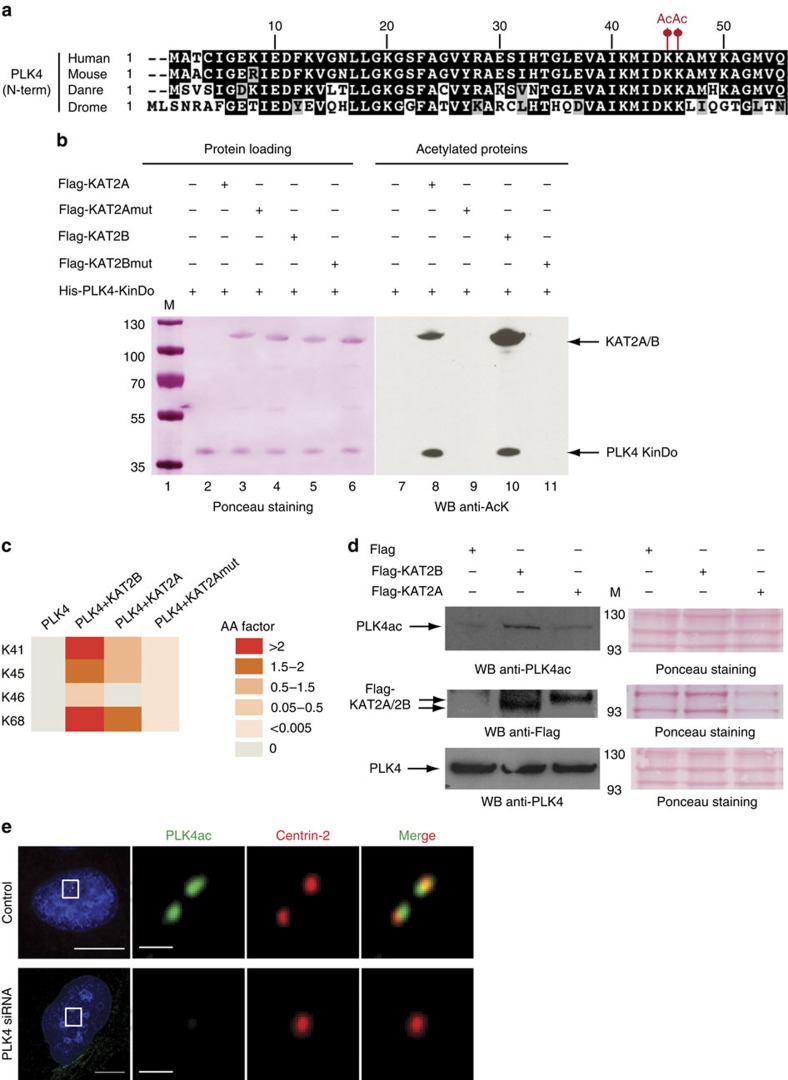
KAT2A and KAT2B acetylate the kinase domain of PLK4. (**a**) Alignment of the first 55 amino acids of human PLK4 with the corresponding sequences from several other species. Danre, *Danio rerio*; Drome, *Drosophila melanogaster*; Human, *H. sapiens*; Mouse, *Mus musculus*. The well-conserved acetylated K45 and K46 residues are highlighted with red dots. (**b**) *In vitro* AT assay on recombinant PLK4. Molecular weight markers are indicated on the left in kDa. (**c**) PLK4 kinase domain (KinDo) was acetylated by KAT2A, KAT2Amut or KAT2B, and the acetylation sites determined after LC–MS/MS analysis. Acetylation abundance factor (see Methods) values are depicted using the indicated colour code. (**d**) HEK293 cells were transfected with expression vectors coding for only the Flag tag, Flag-tagged KAT2A or Flag-KAT2B. Western blots were probed with the indicated antibodies. (**e**) PLK4ac (green) and Centrin-2 localization (red) were probed by immunofluorescence. Scale bars represent 5 μm for the high-magnification view, 1 μm for the inset.

To further analyse the KAT2A/2B-dependent acetylome, we focused on centrosome duplication, a process in which a role for lysine acetylation was not established before our study. Given that PLK4 is one of the key regulators of centrosome duplication, and as the acetylated lysine residues (K45/K46) are localized in the PLK4 kinase domain (see tandem mass spectra of the acetylated PLK4 peptide in Supplementary Data 1 and Supplementary Fig. 2a), we set out to investigate the biological relevance of these acetylations. Note that these two lysine residues are conserved in PLK4 proteins from *Drosophila* to human (Fig. 2a), suggesting functional relevance.

To test whether PLK4 can be acetylated by KAT2A or KAT2B *in vitro*, we performed AT assays on recombinantly expressed PLK4 kinase domain in the presence of acetyl-CoA and purified recombinant human KAT2A or KAT2B (Fig. 2b). As a negative control, we also expressed the recombinant catalytic mutants (mut) of KAT2A and KAT2B. Histone H3 acetylation by KAT2A/2B (ref. 35) was used as a positive control (Supplementary Fig. 2b, lanes 5 and 11). Importantly, we found that recombinant PLK4 kinase domain was acetylated in the presence of purified KAT2A or KAT2B (Fig. 2b, lane 8 and 10). By contrast, the kinase domain was not acetylated when the catalytic dead double point mutant of KAT2A (ref. 36) (lane 9) or KAT2B (lane 11) were used instead. Note, however, that the double point mutant of KAT2B retained some residual acetylation activity towards the histone octamer substrate (Supplementary Fig. 2b, lane 12).

To further validate the acetylation of K45 and K46 residues identified in the comprehensive proteomic analysis, *in vitro* AT assays were performed on recombinant PLK4 kinase domain, followed by tandem MS analysis (Supplementary Fig. 2c and Fig. 2c). Lysines K45 and K46 were found to be acetylated by KAT2A or KAT2B, as were another two lysines of the ATP-binding site (K41 and K68), which were not identified in the large-scale study. Note that the *in vitro* AT assay using purified recombinant protein fragments may result in more sites than the ones identified in cells on endogenous full-length PLK4 protein. The above results together demonstrate that PLK4 can be acetylated by KAT2A/2B on residues K45/K46 in its kinase domain.

To further address whether PLK4 is acetylated in cells, we have developed an antibody against a PLK4 peptide acetylated on positions K45 and K46 (Supplementary Methods and Supplementary Fig. 3a,b). When HEK293 cells were transfected with expression vectors for Flag-KAT2A or Flag-KAT2B, we detected an increase of the acetylated form of PLK4 (PLK4ac), in particular on overexpression of Flag-KAT2B, but not of overall PLK4 levels (Fig. 2d). Moreover, the anti-PLK4ac antibody detected centrosomes in immunofluorescence experiments, with the signal being juxtaposed with that of antibodies directed against the centriolar protein Centrin-2 (ref. 26; Fig. 2d, upper panels). Importantly, the PLK4ac signal completely disappeared when PLK4 was knocked down with siRNAs (lower panel), demonstrating that the raised antibodies are specific to PLK4.

### KAT2A/2B and PLK4ac co-localize at centrosomes

**Figure 3:**
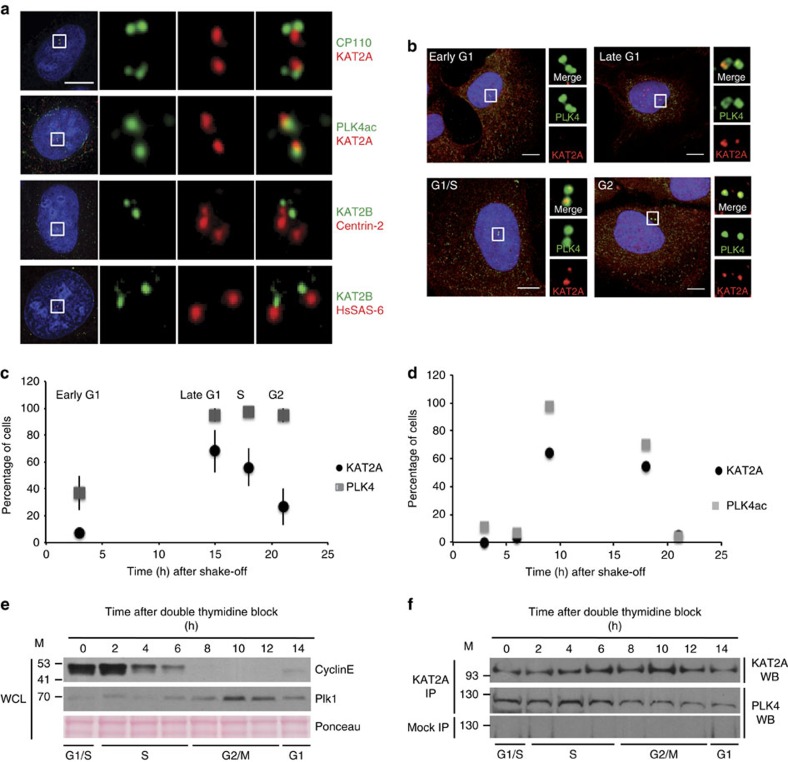
KAT2A/2B and PLK4 co-localize at centrosomes. (**a**) The co-localization of KAT2A (red) or KAT2B (green) with several centriolar proteins, HsSAS-6, Centrin-2, CP110 and PLK4ac was tested by immunofluorescence in U2OS cells. Scale bars represent 5 μm, and insets are high-magnification views of the regions indicated in the low-magnification images. (**b**) PLK4 and KAT2A localization probed by immunofluorescence at different cell cycle stages following synchronization^60^,^61^. U2OS cells were tested by immunofluorescence with antibodies against the kinase domain of PLK4 or KAT2A. (**c**,**d**) The histograms report the average frequency of cells displaying detectable PLK4 and KAT2A (**c**), or PLK4ac and KAT2A (**d**) Signal at centrosomes in different cell cycle stages. Data were collected from three experiments. *n*>100 cells per cell cycle stage. Error bars: s.e.m. (**e**) Cell cycle-staged HeLa cell extracts were prepared. The cell cycle distribution was verified by western blot. (**f**) Using these cell cycle-staged protein extracts IP experiments were carried out with either anti-KAT2A antibodies (KAT2A IP) or control antibodies (mock IP). The presence of KAT2A and PLK4 in the IPs was tested by western blot analysis (WB). M, molecular weight marker in kDa.

Because we identified PLK4 as an acetylated target of KAT2A/2B, and since PLK4 localizes to centrosomes^26^, we investigated whether KAT2A or KAT2B also localize to centrosomes, and this across the cell cycle. To this end, we conducted immunofluorescence analyses using antibodies raised against KAT2A, or against KAT2B, together with antibodies against one of several centriolar proteins: HsSAS-6 (ref. 37); Centrin-2 (ref. 38); CP110 (ref. 39); or else PLK4ac. These experiments showed that both KAT2A and KAT2B could indeed be detected at centrosomes (Fig. 3a).

We then sought to test when during the cell cycle such centrosomal localization occurs, focusing our analysis on KAT2A. This revealed that KAT2A, which is known to localize to the nucleus in interphase and to spindle poles in mitosis^14^, localized also to centrosomes, in particular in late G1 and around the G1/S transition, coinciding with the onset of centriole formation (Fig. 3b,c). PLK4 is enriched at centrosomes throughout the cell cycle and is thought to be critical during late G1 and/or at the G1/S transition to initiate centriole assembly^40^,^41^,^42^. In agreement with the fact that PLK4 is acetylated by KAT2A/2B, we found that the percentage of cells in which PLK4ac and KAT2A were both present at centrosomes was maximal in late G1 and the G1/S transition (Fig. 3d). These observations together suggest that PLK4/PLK4ac and KAT2A/2B co-localize at centrosomes, primarily during late G1 and early S phases.

We next addressed whether KAT2A and PLK4 could associate in cells during late G1 and early S phases. To this end, we first synchronized HeLa Kyoto cells in G1/S using a double thymidine block and then released them for given times before collection (Fig. 3e). Whole-cell protein lysates (WCLs) were prepared and the cell cycle profile verified using the cell cycle progression markers Cyclin E and PLK1 (refs 43, 44; Fig. 3e). Using these staged protein extracts, immunoprecipitation (IP) experiments were carried out with anti-KAT2A antibodies or with control antibodies (Fig. 3f). Western blot analysis using anti-PLK4 and anti-KAT2A antibodies showed that KAT2A co-immunoprecipitated PLK4 mainly during the G1/S transition and S phases, while no PLK4 was detected in the control IP (Fig. 3f). Therefore, our results together suggest that KAT2A/2B and PLK4 co-localize at centrosomes and can associate mostly during late G1 and S phases in human cells.

### KAT2-containing complexes regulate centrosome duplication

**Figure 4:**
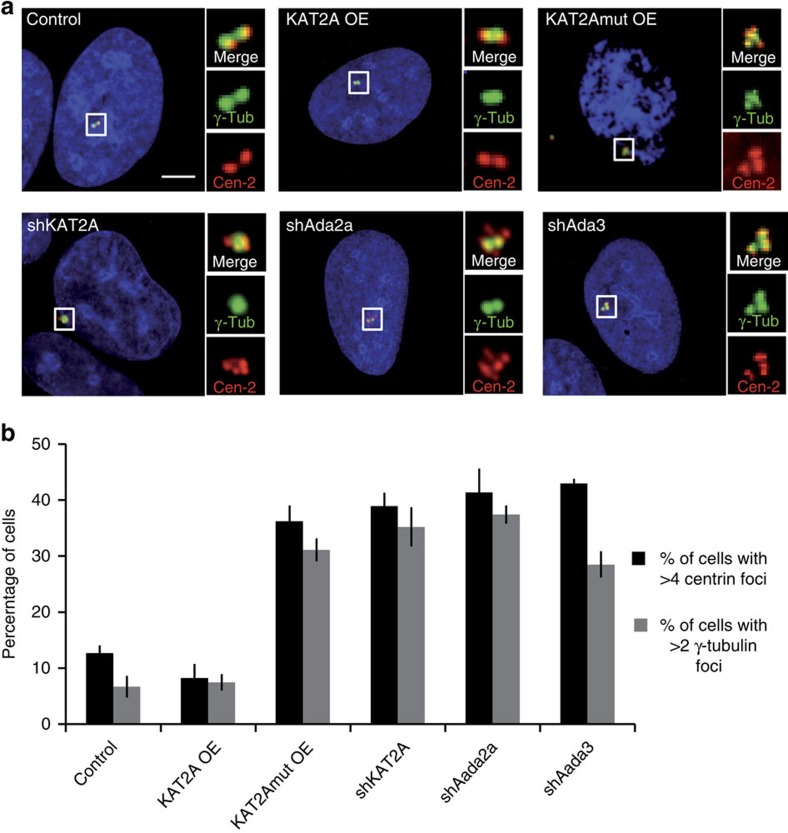
KAT2A and the ATAC complex regulate centrosome duplication. (**a**) U2OS cells were transfected with vectors either to overexpress (OE) or to knockdown the indicated proteins using shRNA-mediated depletion. Centrosomes were visualized by immunofluorescence. Cen-2, Centrin-2; γ-Tub, γ-tubulin. Note that all cells were analysed, without knowledge of their transfection status thus, the values reported here are probably underestimates. (**b**) The histograms report the average frequency of interphase cells displaying aberrant Centrin-2 (>4) and γ-tubulin (>2) numbers. Data from three experiments, *n*=50 cells per condition. Error bars: s.e.m.

Next, we set out to investigate the role of KAT2A/2B in the regulation of centrosome number in human cells. We previously reported that depletion of the ADA2a/ADA3 subunits of the AT module of the ATAC complex results in supernumerary centrosomes in 3T3-NIH fibroblasts^14^, compatible with the idea that KAT2A/2B may be negative regulators of centrosome duplication. However, the underlying molecular mechanism remained elusive. To investigate the hypothesis that KAT2A/2B negatively regulates centrosome numbers through PLK4 acetylation, human U2OS cells were transfected with an expression vector expressing human KAT2A or its catalytic dead double point mutant (KAT2Amut)^36^. Forty-eight hours after transfection, cells were fixed and centrosomes were visualized by immunofluorescence using antibodies against the pericentriolar material component γ-tubulin and the centriole component Centrin-2 (Fig. 4a,b). Whereas cells overexpressing the wild-type (WT) version of KAT2A did not experience alterations in centrosome number, we found that cells overexpressing KAT2Amut had supernumerary centrosomes, suggestive of a dominant negative effect (Fig. 4b). Moreover, we found that shRNA-mediated depletion of KAT2A, as well as that of ADA2a or ADA3, which are all subunits of the AT module of ATAC complex^22^, led to a similar phenotype as that of cells overexpressing KAT2Amut (Fig. 4a,b). Note that the knockdown of ADA3 depletes the AT activity of both KAT2A- and the KAT2B-containing ATAC complexes^22^. Together, these results indicate that KAT2A/2B AT activity in the ATAC coactivator complexes plays an essential role in restricting centrosome duplication in human cells.

### Acetylation of PLK4 stabilizes its inactive conformation

**Figure 5:**
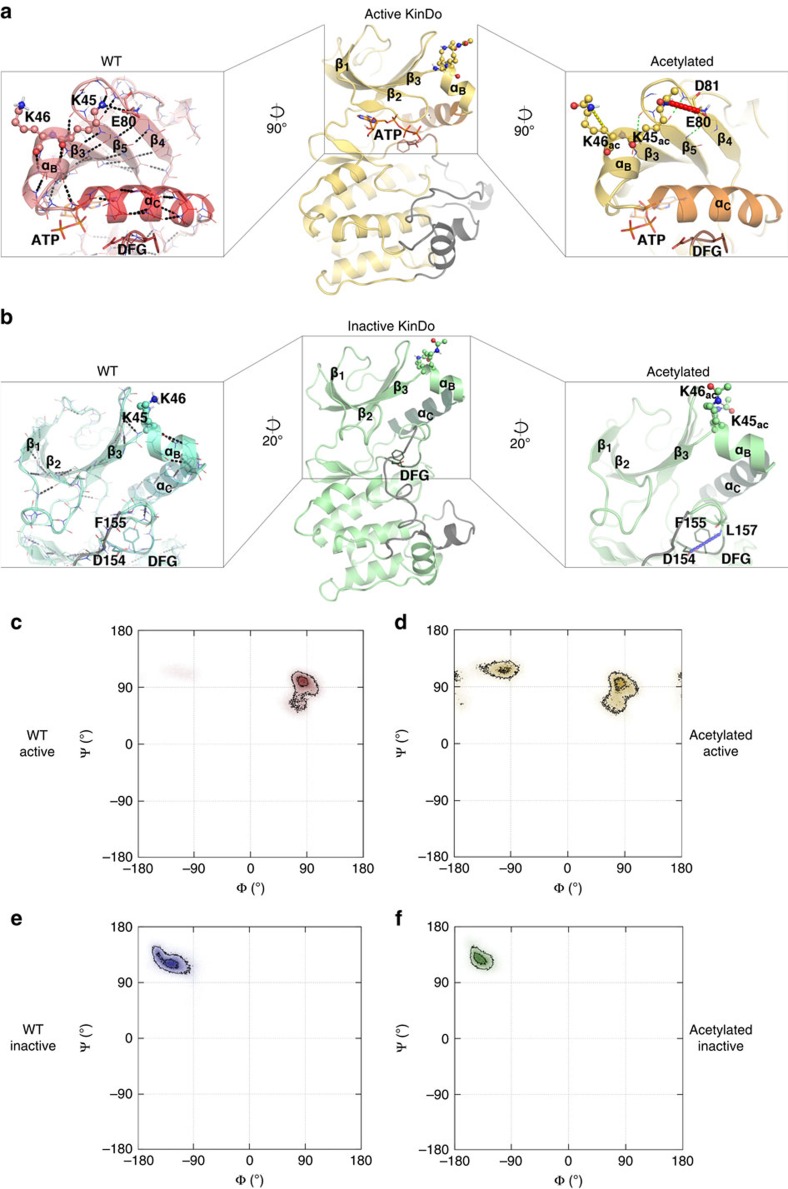
Molecular modelling of PLK4 kinase domain suggests that acetylation shifts the dynamics of the acetylated PLK4 to the inactive conformation. (**a**) Active (PDB ID 3COK) and (**b**) inactive (PDB ID 4JXF) conformations of the kinase domain (KinDo) of PLK4 are shown in the middle of the panels. The region where the structure was reconstructed is shown in grey. In both conformations, zoomed-in views have been generated to highlight the changes caused by K45/K46 acetylation. Zooms on the left show the non-modified WT KinDo structures, zooms on the right the KinDO structures with K45/K46 acetylations (ac). The zooms are rotated with the indicated angles when compared with the structures in the middle. On the right, the atoms of the backbone are included. Dotted lines are plotted from nitrogen atoms to oxygen atoms. The left side zooms give an overview of the most stable H-bonds of the WT KinDo, while the right side zoom only shows the H-bonds that differ in the acetylated forms. The thicker lines in **a** show the local H-bonds that are lost on acetylation: K45-E80 (red) and K46-D44 (yellow), whereas weakened H-bonds are shown in green. If an H-bond is not reported from left to right, its stability does not vary. In **b**, a distant H-bond newly formed in the acetylated structure (D154-L157) is highlighted in blue. The ATP and residues of the DFG motif are shown in sticks, whereas the αC helix and other structural motifs are in ribbons. (**c**–**f**) Ramachandran representation of the backbone dihedral angles of D154 (of the DFG motif), in the WT (**c**,**e**) and acetylated (**d**,**f**) forms of the active and inactive conformations. (**d**) In the active acetylated form, D154 explores conformations characteristic of the inactive structure, whereas this is not observed for the WT (**c**).

Dynamic switching between active and inactive conformations regulates kinases so that their inhibition can be achieved by trapping them in their inactive state^45^,^46^. Scrutiny of the PLK4 kinase domain revealed that K45 and K46 are located at the N-terminal extremity of α-helix B, a short helix situated N-terminal and almost perpendicular to the main regulatory α-helix C of the kinase (Fig. 5a,b). Thus, acetylation of K45 and K46 residues by KAT2s could conceivably affect the structure of the kinase domain and/or its dynamic equilibrium between active/inactive conformations. To address these possibilities and to understand the consequences of K45 and K46 acetylation, we carried out molecular dynamics simulations of the kinase domain. These *in silico* experiments revealed that such acetylation is expected to directly affect the H-bond network of K45 and K46, thus suppressing their interactions with neighbouring charged residues and weakening a few backbone–backbone H-bonds within the N-lobe β-sheets of the kinase domain (Fig. 5a,b).

The modelling uncovered also that K45/K46 acetylation might alter the structural dynamics of the kinase domain in regions that are somewhat distant from the acetylation site. Of particular interest, differences were noted in interaction networks and residue-to-residue distances, or dihedral angles, which involve amino acids of the kinase domain important for catalytic activity. The modelling indeed established differences in the inactive conformation of the β1–β2 loop, which is part of the ATP-binding site. Thus, the acetylated version of the protein adopts a different conformation, in which van der Waals interactions are reinforced in this loop, which may tilt the balance towards the inactivate state. Furthermore, the most sizeable differences due to K45/K46 acetylation are observed around the D_154_F_155_G_156_ (DFG) motif of the activation loop, which is central for kinase activation by ensuring transitions between active and inactive structures^46^,^47^. Moreover, backbone dihedral angles of residues A153 and L157 were affected as well. Importantly, the distribution of phi/psi angles sampled by D154 of the DFG loop differs in the WT and acetylated simulations (Fig. 5c–f), additionally suggesting that acetylation could stabilize the inactive conformation of the kinase. In conclusion, the simulation data indicate that K45/K46 acetylation would not only affect structural networks locally, but also at more distant amino acids, which are crucial for kinase catalytic activity. Moreover, these data indicate that such modifications are expected to shift the dynamics of the acetylated PLK4 towards the inactive conformation.

### Acetylation of PLK4 by KAT2A/2B inhibits its kinase activity

**Figure 6:**
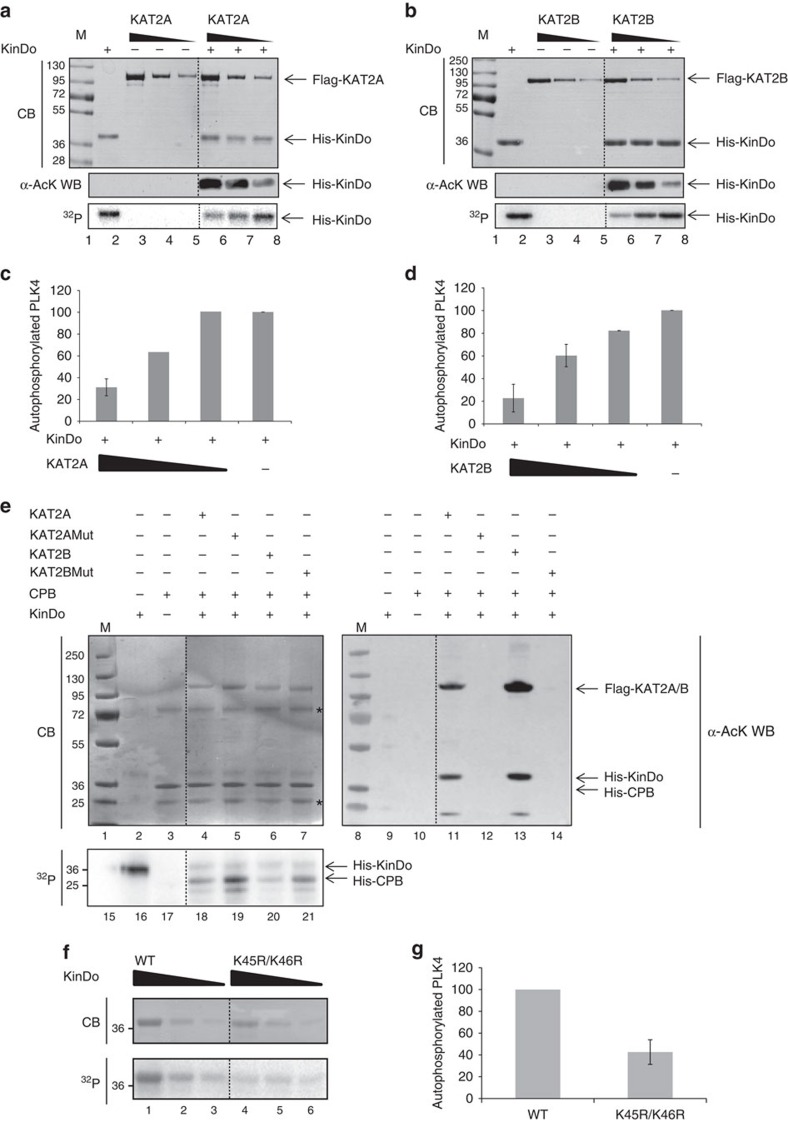
Acetylation of PLK4 kinase domain by KAT2A/2B inhibits its kinase activity. (**a**,**b**) Kinase activity of the PLK4 kinase domain (KinDo) in the presence of ^32^Pγ-ATP after being acetylated by Flag-KAT2A (**a**) or Flag-KAT2B (**b**). KinDo acetylation was tested by western blot analyses (WB). Kinase activity was measured by the autophosphorylation reaction (^32^P) of the KinDo. M, molecular weight marker in kDa. (**c**,**d**) Kinase activity normalized to protein amount was measured in three independent experiments. Mean values from the three replicate experiments and the corresponding s.d.'s are shown. (**e**) Kinase activity of the KinDo was tested on His-CPB of PLK4. KinDo was first acetylated by Flag-KAT2A, Flag-KAT2Amut, Flag-KAT2B or Flag-KAT2Bmut and then ^32^Pγ-ATP was added to the reactions. The level of acetylated KinDo was tested by WB. Kinase activity was measured by phosphorylation (^32^P) of His-CPB by the KinDo. M, molecular weight marker in kDa. (**f**) Kinase activity of the recombinant purified WT or mutated (K45R/K46R) KinDos. Protein levels and autophosphorylation activities were analysed as in **a**,**b**. (**e**) The kinase activities of WT and mutated K45R/K46R KinDos were normalized and measured from three independent experiments as in **c**,**d**. In **g**, kinase activity of the K45/K46R KinDo was calculated relative to that of the WT KinDo, which was normalized to 100% in all replicates. In **a**,**b**,**e**,**f**, the reaction mixes were analysed by Coomassie blue (CB) staining. In **a**,**b**,**e**,**f**, a dotted line has been inserted to indicate where unnecessary lanes were cut out from the gels.

To test the above structural predictions, we set out to determine the influence of acetylation on the kinase activity of the PLK4 kinase domain. Kinase assays were performed with either unmodified or by KAT2A- or KAT2B-acetylated kinase domain (Fig. 6a–d). Acetylation of the PLK4 kinase domain in these experiments was tested by western blot using an anti-pan-acetyl-lysine antibody, whereas autophosphorylation of the recombinant kinase domain was quantified as a readout of PLK4 kinase activity^28^. We found that acetylation of the PLK4 kinase domain by either KAT2A or KAT2B significantly reduced PLK4 kinase domain activity (Fig. 6a–d), with the extent of inhibition inversely correlating with the strength of acetylation (Fig. 6a–d). Thus, in agreement with the above modelling data, acetylation of PLK4 has an inhibitory effect on its kinase activity.

We tested KAT2A- or KAT2B-acetylation-dependent inhibition of PLK4 kinase activity using a different substrate. To this end, we expressed and purified the crypto polo box (CPB) domain of PLK4, another substrate of PLK4 (ref. 30). The kinase domain was pre-acetylated by KAT2A or KAT2B in the presence of CPB, and the phosphorylation of CPB and PLK4 then detected by autoradiography (Fig. 6e, see ^32^P panel). The effect of the acetylation on the kinase domain was also verified by using the catalytic dead mutants of KAT2A and KAT2B (Fig. 6e and Supplementary Fig. 2b). We found that the presence of either KAT2A or KAT2B AT activity dampened PLK4 kinase domain activity as measured by CBP phosphorylation (Fig. 6e). These results confirm that acetylation of PLK4 inhibits its kinase activity.

Next, we investigated whether lysines K45 and K46 are important for regulating PLK4 kinase activity. K to Q replacements mimic acetylation by substituting the charged lysine by a neutral analogue bearing an amide side chain, although it has been suggested also that this may overestimate the impact of this post-translational modification^48^. Moreover, our molecular dynamics simulations of the PLK4 kinase domain containing K45Q and K46Q replacements revealed that the mimic would be imperfect in this case, since the Q side chains are significantly shorter than those of the acetylated K. As a result, local perturbations of the H-bond network around the mutation sites, both in the active and in the inactive conformations, were found in the catalytic site of the kinase domain (Supplementary Fig. 4c,d), in contrast to the simulations obtained with acetylated K45/K46, which shifted the equilibrium towards the inactive conformation (Fig. 5). As an alternative means to test whether lysines K45 and K46 are important for regulating PLK4 kinase activity, we generated a mutant version in which K45 and K46 are replaced with non-acetylable arginine (R) residues. Interestingly, molecular dynamics simulations suggested that these substitutions stabilize the kinase in its inactive conformation without influencing its general structure (Supplementary Fig. 4a,b). In spite of the fact that the K45R/K46R mutant cannot be acetylated, the modelling suggested that the arginine substitutions may stabilize the kinase in its inactive conformation similarly (although weaker) as do the acetylations of the K45 and K46 residues in the WT kinase (Supplementary Fig. 4a,b). Accordingly, we found that the kinase activity of the K45R/K46R mutant kinase domain was reduced 2.5-fold when compared with the WT (Fig. 6f,g).

Although we cannot formally exclude that the impact of the K45R/K46R mutations might not only influence the acetylation of K45/K46, but also impair the kinase activity irrespective of these modifications, these results taken together indicate that *in vitro* acetylation of PLK4 at positions K45 and K46, or mutation of these residues to R45/R46, inhibits PLK4 kinase activity.

### KAT2A/2B are required for proper phosphorylation of PLK4

**Figure 7:**
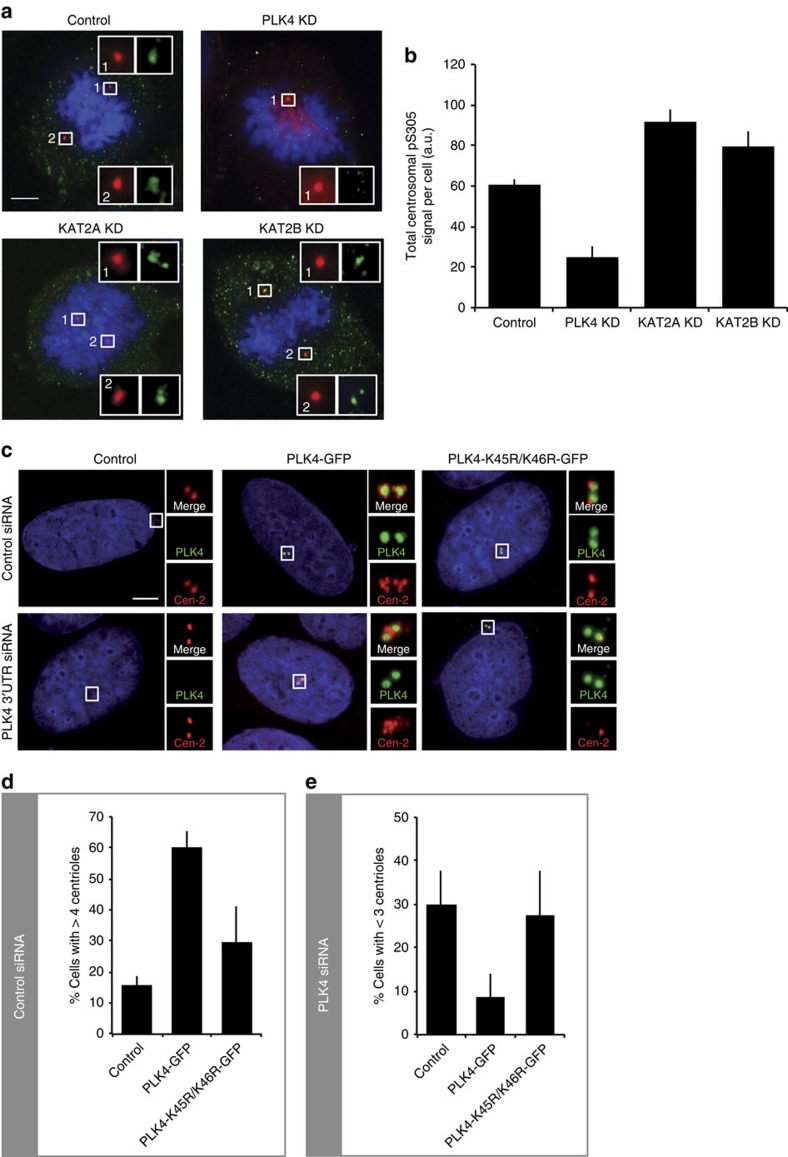
KAT2A/2B-mediated acetylation of PLK4 prevents abnormal centrosome amplification in human cells. (**a**) Distribution of PLK4 phosphorylated at Ser305 (pSer305) upon KAT2A/2B knockdown. U2OS cells were transfected with control siRNAs (control), or siRNAs targeting PLK4, KAT2A or KAT2B to knock down (KD) these factors. Cells were stained with anti-PLK4-pS305 antibodies (green) and γ-tubulin (red). Scale bar represents 5 μm, and insets are high-magnification views of the regions indicated in the low-magnification images. (**b**) Quantification of total centrosomal pS305 signal in cells of the indicated conditions (reflected in the images shown in **a**). *n*=10 cells for each condition. Student's two-tailed paired *T*-test (comparing with WT): KAT2A depletion <0.001; KAT2B depletion 0.038. Note that we found no statistically significant increase on KAT2A or KAT2B depletion if each focus of PLK4-pS305 was considered instead of the total centrosomal signal per cell. (**c**) U2OS cells were transfected with control siRNAs (upper row), or siRNAs targeting the 3′-untranslated region (UTR) of *PLK4* (PLK4 3′-UTR siRNA, lower row). Twenty-four hours later, cells were transfected again with control-empty vectors, a vector encoding the full-length PLK4-GFP, or a vector encoding the full-length PLK4-K45R/K46R-GFP double point mutant. Cells were stained for GFP (green) and Centrin-2 (Cen-2). (**d**) The histogram reports the average frequency of mitotic cells displaying >4 centrioles in the siRNA control condition. (**e**) The histogram reports the average frequency of mitotic cells displaying <3 centrioles in the siPLK4 3'-UTR condition. Data from three experiments, *n*=50 cells per condition. Error bars: s.e.m.

Next, we investigated whether KAT2A or KAT2B are necessary for proper phosphorylation of endogenous PLK4, which provides a read-out of PLK4 activity^40^. To this end, we have carried out siRNA knockdown experiments of either KAT2A or KAT2B, detecting PLK4 auto-phosphorylation by immunofluorescence using an antibody detecting PLK4 phosphorylated at position Ser305 (pSer3005)^40^. In parallel, centrosomes were marked with antibodies directed against γ-tubulin. Cells in mitosis were quantified for the extent of total centrosomal pSer305 signal. Importantly, these experiments revealed that PLK4 phosphorylation augmented when KAT2A or KAT2B were knocked down (Fig. 7a,b). By contrast, levels of total centrosomal PLK4 appeared not to be altered (Supplementary Fig. 5). These results suggest that KAT2A/2B are required for proper phosphorylation of PLK4 in human cells.

### PLK4 acetylation by KAT2s prevents centrosome amplification

To analyse the cellular role of PLK4 acetylation in regulating centrosome numbers, we generated full-length PLK4-GFP vectors with either K45/K46 (WT) or K45R/K46R (double mutant). Cells were transfected with these expression vectors and examined 48 h thereafter by immunofluorescence with antibodies against green fluorescent protein (GFP), to monitor the localization of the fusion proteins, as well as against Centrin-2, to mark centrioles. We noted first that both WT and K45R/K46R mutant version localized to centrosomes (Fig. 7c). Second, the number of Centrin-2 foci in mitotic cells was determined as read-out of successful centriole formation. Cells transfected with the empty vector (control) usually harboured four centrin foci in mitosis, with only ∼16% of cells having more than four (Fig. 7c,d). As reported previously^26^, overexpression of WT PLK4-GFP caused massive centriole amplification, with ∼60% of mitotic cells having more than four centrioles (Fig. 7c,d). By contrast, overexpression of PLK4-K45R/K46R-GFP only mildly increased the percentage of cells with more than four centrioles (∼30% of cells; Fig. 7c,d and Supplementary Fig. 6a). Moreover, the centriole amplification provoked by overexpression of WT PLK4-GFP was dampened by the concomitant overexpression of KAT2A or KAT2B (Supplementary Fig. 6b). In addition, as anticipated, removal of PLK4 activity using either siRNAs or the small molecule centrinone also prevented centriole formation in cells that were depleted of KAT2A or KAT2B in addition (Supplementary Fig. 6c). Taken together, these results demonstrate that the combined point mutations in the kinase domain severely impair PLK4 function.

Next, endogenous PLK4 was depleted using siRNAs targeting the 3′-untranslated region of the genomic *PLK4* locus, a region that is absent from the two PLK4-GFP (WT and double mutant) expression vectors. Depletion of endogenous PLK4 by siRNA caused centriole underduplication, as evidenced by the fact that ∼30% of cells contained less than three centrioles during mitosis (Fig. 7e). Expression of WT PLK4-GFP rescued this phenotype. In contrast, expression of PLK4-GFP[K45R/K46R] did not (Fig. 7e), further demonstrating that this mutant is inactive. Thus, our *in vitro* and *in vivo* experiments concur to suggest that AT activities of KAT2A/2B and acetylation of K45/46 in PLK4 are necessary to restrict the number of centrosomes.

## Discussion

The role for acetylation in the regulation of several cellular processes is well documented. Moreover, the widespread nature of protein acetylation and the large number of identified acetylated proteins (more than 6,000)^10^ raised questions about the role of individual KATs and their importance in regulating different cellular functions. Here we describe the identification of about 400 proteins acetylated by KAT2A and KAT2B. Gene ontology analyses indicated that these acetylated non-histone proteins play a role in well-defined cellular functions, such as chromatin remodelling, transcriptional regulation, DNA replication, DNA repair, cell death and cell cycle progression. KAT2A and 2B were already suggested to be involved in several of these processes (see Introduction). Interestingly, here we identified acetylated proteins involved in novel pathways, such as actin-mediated cell contraction, protein transport, phosphorylation and regulation of centrosome duplication (Fig. 1 and Supplementary Data 1), suggesting that by acetylating these non-histone proteins KAT2A/2B can also regulate their function. It is tempting to speculate that the coordinated acetylation by KAT2A/2B of histones and non-histone proteins at regulatory genomic locations could be linked. Indeed, by loosening the chromatin at their site of action, KAT2A/2B could facilitate the recruitment of factors involved in the chromatin-associated processes mentioned above and modulate their action by further acetylation.

Other kinases besides PLK4 may be acetylated by KAT2A/2B, including CDK2 (ref. 49), CDK5 (ref. 50) and CDK9 (ref. 51). However, the mechanism of kinase inhibition is different in those cases when compared with PLK4. Indeed, KAT2A/2B acetylate CDK2, CDK5 and CDK9 at the ATP-binding sites (located at positions K33 of human CDK2 and CDK5, and at position K48 of human CDK9 (Supplementary Fig. 7). The impact of acetylation on the ATP-binding site of CDK5 has not been studied. However, mutations of the ATP-binding sites into non-acetylable residues (R) in CDK2 and CDK9 inhibited kinase activity by preventing ATP binding^49^,^51^. Importantly, our results suggest a different mechanism through which acetylation inhibits kinase activity in the case of PLK4. Indeed, our molecular modelling experiments indicate that acetylation not only affects structural networks near the acetylated lysines, but also at more distant amino acids, which are crucial for kinase catalytic activity (such as the DFG motif). Importantly, in addition, the simulations suggest that these modifications shift the dynamic equilibrium of the acetylated PLK4 towards its inactive conformation. Thus, our data suggest that KAT2A/2B-dependent acetylation of PLK4 in the kinase domain is required to limit kinase activity of PLK4, perhaps primarily during S phase after PLK4 has acted to initiate centriole formation, to avoid subsequent rounds of centriole formation and centrosome overamplification. KAT2A/2B are therefore important for the maintenance of genomic stability.

The serine/threonine PLK4 is a pivotal regulator of centrosome duplication. Identified PLK4 substrates include Cep152 (ref. 52) and STIL^53^, the modifications of which are both critical for the onset of procentriole assembly^52^,^53^ In this study, we have uncovered that centrosome duplication in mammalian cells is modulated by acetylation of PLK4. Our data indicate that acetylation negatively regulates PLK4 kinase activity and thus prevents centrosome overamplification. This is in line with previous reports showing that lysine acetylation is detected at centrosomes by immunofluorescence using a pan-acetyl lysine antibody^54^. While we have described a role for the acetyltransferases KAT2A/2B in the regulation of centriole numbers, other studies have reported roles for lysine deacetylases (KDACs) in regulating centrosome function. For example, it was shown that KDAC8 depletion enhances centrosome splitting in human cells, while KDAC1 depletion or treatment with the KDAC inhibitor trichostatin A had opposite effects, with reduced centrosome splitting^55^. Moreover, it has been proposed that proteins involved in centrosome cohesion would be acetylated and substrates of these KDACs. This suggestion was further supported by localization studies showing that several KDACs, including KDAC1, localize at centrosomes^54^. The above findings notwithstanding, the exact mechanism for the regulation of centrosome architecture and/or numbers by KDACs is unclear.

The activity and stability of PLK4 have to be tightly controlled to avoid severe defects, such as genomic instability^23^,^56^. The mechanisms governing PLK4 protein stability have been well studied, with PLK4 regulating its own stability by autophosphorylation, which triggers ubiquitin-mediated proteasomal degradation (reviewed in refs 28, 29, 30). However, the mechanisms controlling PLK4 kinase activity have remained elusive. In this study, we show that lysine acetylation regulates PLK4 kinase activity. It is conceivable that degradation of PLK4 and acetylation-dependent inhibition of kinase activity act in concert to achieve a tight control of centrosome duplication. In the future, it will be interesting to investigate whether these PTMs act independently or in a coordinated fashion during cell cycle progression to accurately regulate PLK4 function.

## Methods

### Plasmids

The bacterial expression vector petHis30a-HisPLK4 KinDo was a generous gift from A. Holland^29^. The mammalian expression vector pcDNA3-FlagPLK4 has been described^57^. The bacterial expression vector petHis15b-CPB was created by insertion of the PLK4 cDNA fragment aa 635–878 containing the CPB domain into pet15bHis. The PCR fragment was amplified with primers 5′-AGCAGCGGCCTGGTGCCGCGCGGCAGCCATATGGAAGTTCTTCAGATATCTAGTGAT-3′and 5′-TCGGGCTTTGTTAGCAGCCGGATCCTCATTTTAGACTATTAGAAGAGA-3′, and then cloned by recombination into the NdeI/BamHI site of the pET15b vector. The baculovirus expressing Flag-tagged KAT2A (GCN5) and the expression vector for Flag-tagged KAT2A (GCN5) catalytic mutant in which E575 and D615 were both replaced by alanine were described in ref. 14. The Flag-tagged KAT2B (PCAF) baculovirus expression vector was kindly provided by N. Rochel-Guiberteau. The baculovirus expression vector for Flag-tagged KAT2B (PCAF) catalytic mutant in which E570 and D610 were both replaced by alanines was made by insertion of a PCR fragment containing the mutations. The DNA was amplified with primers 5′-GGAAGATCTCCACCATGGATTACAAGGATGACGACGATAAGCCCGGGTCCGAGGCTGGCGGGGCCGGGCCG-3′ and 5′-CGCGAATTCTCACTTGTCAATTAATCCAGCTTCCTT-3′, digested by BglII and EcoRI, and ligated into pVL1393 baculovirus expression vector digested with BglII and EcoRI. The mammalian expression vector pcDNA3-Flag and pcDNA3-FlagKAT2A have been described in ref. 14. The mammalian expression vector pCDNA3-FlagKAT2B was provided by the Addgene plasmid repository.

K to R point mutations at K45 and K46 in the PLK4 kinase domain were introduced within the petHis30a-HisPLK4KinDo or the hPLK4-EGFP-pCDNA plasmids by PCR direct-site mutagenesis using the following primers: 5′-GCAATCAAAATGATAGATCGACGAGCCATGTACAAAGCAGGA-3′ forward; and 5′-TCCTGCTTTGTACATGGCTCGTCGATCTATCATTTTGATTGC-3′ reverse. The primers were used for amplification of the template DNA. After 15 cycles using Pfu DNA polymerase, the product was treated with DpnI and then transformed directly into *Escherichia coli* XL1 Blue. Miniprep plasmid DNA was used to sequence and control the point mutations.

### Antibodies

Antibodies against KAT2A (GCN5; mouse monoclonal antibody (mAb) 2GC2C11, dilution 1:1,000^58^; or mAb 5GC2A6 (ref. 14), dilution 1:1,000), KAT2B (PCAF; antibody, Santa Cruz sc-13124; sc-8999; dilution 1:500 for immunofluorescence studies), rabbit acetyl histone H3 (Milipore 06-599, dilution 1:2,000), rabbit pan acetyl-lysine rabbit polyclonal (Cell Signaling, #9441, dilution 1:1,000), γ-tubulin (mAb, Sigma-Aldrich GTU-88; dilution 1:1,000), rabbit polyclonal γ-tubulin (Sigma-Aldrich, T 5192, dilution 1:1,000), rabbit monoclonal PLK4 (Abcam #Ab109008, dilution 1:500), Centrin-2 (mAb, Merck-Millipore 20H5; dilution 1:4,000), rabbit polyclonal PLK4-pS305P ((ref. 40), dilution 1:500, gift from M. Bornens, Institut Curie, Paris, France), rabbit GFP (dilution 1:500, gift from V. Simanis, Swiss Federal Institute of Technology, Lausanne, Switzerland), HsSAS-6 (mAb, Santa Cruz sc-81431; dilution 1:500), rabbit polyclonal CP110 (ProteinTech, 12780–1-AP; dilution 1:1,000), Flag (mAb, Sigma-Aldrich F1804, clone M2; dilution 1:1,000), Flag M2 affinity gel, A2220 (Sigma-Aldrich), rabbit CyclinE (Santa-Cruz sc-481, M-20; dilution 1:1,000), Plk1 (mAb, Sigma-Aldrich 36-298 #P6123; dilution 1:1,000) have been described previously or have been purchased as indicated.

To generate polyclonal antibodies against PLK4ac, a peptide acetylated at positions K45 and K46 was synthesized: from aa 34 (TGLEVAIKMIDKacKacAMYKAGMVDQR-C) to aa 56. The same peptide without the acetylated residues was also synthetized. The acetylated peptide was coupled to ovalbumin carrier protein and used for immunization of rabbits at the IGBMC rabbit facility. Collected sera were first purified on a SulfoLink column (Pierce) to which the peptide that did not contain the acetylated residue has been conjugated through its C-terminal cysteine. Affinity columns were prepared as specified by the manufacturer. After having passed the sera through the non-acetylated peptide-containing column five times, the flow-through fraction was further purified on an affinity column to which the acetylated peptide had been conjugated. Bound proteins were extensively washed, antibodies were eluted with Tris-Glycine (pH 2.8) buffer, neutralized immediately with 2 M Tris-HCl (pH 8.8), dialysed and kept at −20 °C. Enzyme-linked immunosorbent assay tests were carried out using standard protocols. The rabbit PLK4ac antibody was used at a 1/500 or at 1/5,000 dilutions in the different applications (as indicated).

### Inducible HeLa shRNA cell lines

Generation of the inducible HeLa cells harbouring shRNA directed either against GCN5/KAT2A (target sequences: 5′-CGTGCTGTCACCTCGAATGA-3′) or GL2 luciferase as control (target sequences: 5′-CCTTACGCTGAGTACTTCGA-3′) were described previously in ref. 14.

### Cell synchronization experiments

Cell synchronization experiments were performed by double thymidine block using HeLa Kyoto cells as described in ref. 59. Briefly, cells were first blocked in G1/S with 2 mM thymidine treatment for 18 h, released in fresh media for 9 h and then blocked again in G1/S with 2 mM thymidine treatment for 16 h. After double thymidine block, cells were release in fresh media and collected at 0, 2, 4, 6, 8, 10, 12 and 14 h thereafter. Mitotic shake off was used to synchronize cells in G2/M^60^,^61^.

### Cell extracts and protein purification

For protein purification from baculovirus/insect cells expression system: SF9 cells were infected with baculoviruses expressing Flag-tagged hKAT2A, hKATAmut, hKAT2B or hKAT2Bmut. Forty-eight hours post infection, cells were collected by centrifugation at 1,500 r.p.m. at 4 °C, washed into 1 × phosphate buffer saline solution (PBS1 ×) and lysed into 0.4 M KCl, 15 mM Tris-HCl (pH 8.0), 20% glycerol, 5 mM MgCl_2_, 0.4% NP-40, 1 × protease inhibitor cocktail (Roche) and 1 mM dithiothreitol (DTT). After 10 min of centrifugation at 8,000 r.p.m. at 4 °C the supernatant containing whole-cell protein extracts was collected and incubated with Flag-M2 for IP of Flag-tagged proteins. After 1 h of incubation at 4 °C on a rotating wheel, beads were washed twice with IP buffer (25 mM Tris-HCl (pH 8.0), 10% glycerol, 5 mM MgCl2, 0.1% NP-40, 1 × protease inhibitor cocktail and 1 mM DTT) containing 0.5 M KCl and twice with an IP buffer containing 0.1 M KCl, before elution with a Flag tag peptide DYKDDDDK at 2 mg ml^−1^ final concentration dissolved in 0.1 M KCl IP buffer. Wild-type, mutant recombinant PLK4 kinase domain and recombinant PLK4 CPB domain were purified from *E. coli* (strain Rosetta DE3) as described in ref. 29. To generate whole-cell protein extracts of HeLa cells knocked down for KAT2A and KAT2B, cells were lysed with a buffer containing 0.4 M KCl, 20% glycerol, 20 mM Tris-HCl (pH 7.5), 1 × protease inhibitor cocktail (PIC) and 2 mM DTT, in the presence of 5 mM sodium butyrate (NaB) to preserve acetylated sites, after three cycles of freezing and thawing steps in liquid N_2_. After centrifugation for 30 min at 14,000 r.p.m. at 4 °C, supernatants containing soluble whole-cell protein extracts were collected for further proteomic analyses. To generate whole-cell protein extracts from HEK293 cells overexpressing Flag-KAT2A or Flag-KAT2B, cells were lysed in a buffer containing 150 mM NaCl, 50 mM Tris-HCl, 2 mM EDTA, 0.5% NP-40, benzonase (125 U ml^−1^), 2 mM MgCl_2_, 1 mM DTT, 1 × protease inhibitor cocktail (Sigma), 5 mM NaB as a deacetylase inhibitor and 20 mM sodium fluoride, 20 mM β-glycerophosphate and 1 mM sodium orthovanadate as phosphatase inhibitors. After centrifugation for 30 min at 13,200 r.p.m. at 4 °C, supernatants containing WCL were collected.

For IP of endogenous KAT2A, synchronized, HeLa Kyoto cells were lysed in a buffer containing 150 mM NaCl, 50 mM Tris-HCl, 2 mM EDTA, 0.5% NP-40, benzonase (125 U μl^−1^), 2 mM MgCl_2_, 1 mM DTT, 1 × protease inhibitor cocktail (Sigma), 5 mM NaB as a deacetylase inhibitor and 20 mM sodium fluoride, 20 mM β-glycerophosphate and 1 mM sodium orthovanadate as phosphatase inhibitors. After centrifugation for 30 min at 13,200 r.p.m. at 4 °C, supernatant containing WCL was collected and incubated for 1 h on a rotating wheel at 4 °C, with anti-KAT2A antibodies crosslinked to protein-G agarose magnetic beads. After washing beads with a buffer containing 150 mM NaCl, 50 mM Tris-HCl (pH 8.0), 2 mM EDTA, 2 mM MgCl_2_, 0.1% NP-40, 1 mM DTT, 1 × final protease inhibitor cocktail, 5 mM NaB, 20 mM sodium fluoride, 20 mM β-glycerophosphate and 1 mM sodium orthovanadate, KAT2A immunoprecipitated proteins were eluted from beads in lithium dodecyl sulphate buffer supplemented with 100 mM DTT for 10 min incubation at 85 °C before SDS–PAGE and western blot analysis. Mock IP was performed as described above, but using protein-G agarose beads only.

### Western blot assays

For western blot analyses, protein samples were separated by SDS–PAGE and transferred onto nitrocellulose membrane (Whatman), blocked with 5% skimmed milk in PBS and incubated with primary antibodies as described above. The membranes were then incubated with anti-mouse or -rabbit horseradish peroxidase-linked secondary antibodies (at 1:10,000 dilution) and proteins detected by chemiluminescence using Detection Reagents 1 and 2 (Thermo Scientific).

Uncropped scans of the gels and blots shown in Figs 1a, 2, 3 and 6 are provided as Supplementary Fig. 8

### AT assays

AT assays on recombinant proteins were performed by incubating, for 1 h at 30 °C, His-tagged purified PLK4 kinase domain with purified recombinant Flag-tagged KAT2A, Flag-KAT2Amut, Flag-KAT2B or Flag-KAT2Bmut in the presence of cold acetylcoenzyme A. The reaction buffer contained 50 mM M Tris-HCl (pH 8.0), 10% glycerol, 100 mM EDTA, 50 m M KCl, 0.1 M NaB, 1 × protease inhibitor cocktail (Roche, France) and 5 mM DTT. Reactions were incubated for 1 h at 30 °C, stopped by adding Laemmli buffer with 10% b-mercaptoethanol, boiled for 5 min and loaded on acrylamide gels. The protein mixtures were resolved by SDS–PAGE and analysed by western blot using anti pan-acetyl lysine antibodies after Ponceau staining of the membrane to reveal overall protein distribution.

### Phosphorylation assays using the kinase domain of PLK4

Kinase assays were performed on recombinant PLK4 kinase domain or on the CPB of PLK4 as described in ref. 29. The level of phosphorylation activity of the kinase domain (in the presence of [^32^Pγ]-ATP) was quantified either by autoradiography or by direct counting of the radioactive signal.

### Cell culture and plasmid transfection experiments

HEK293T and HeLa Kyoto cells were grown in Dulbecco's modified medium (DMEM) with 1 g l^−1^ glucose, supplemented with10% fetal calf serum and 100 units per ml penicillin+100 μg ml^−1^ streptomycin (Gibco). U2OS cells were obtained from the EACC and maintained in DMEM (Gibco) containing 1 g l^−1^ glucose, supplemented with 10% fetal bovine serum and gentamycin 40 μg ml^−1^.

DNA and siRNA transfection were performed using Lipofectamine 2000 (Thermo Scientific) or RNAiMax and OptiMEM (Invitrogen), respectively, according to the manufacturers' protocols, and cells were analysed 48–72 h after siRNA treatment. shRNA (for Ada2a, Ada3 and GCN5) and cDNA (for Ada2a, Ada3, GCN5 WT or mutated on the HAT domain) have been previously described in ref. 14. Simultaneous knockdown and overexpression: endogenous PLK4 was depleted using a Stealth siRNA (Invitrogen) targeting the 3′-untranslated region of PLK4 (5-AATAACTTACCAGTAAACTCACTTT-3). Stealth siRNA negative control LO GC (Invitrogen) was used as a control. Twenty-four hours after siPLK4 transfection, cells were transfected with plasmids encoding WT or K45R/K46R PLK4. Forty-eight hours after DNA transfection, cells were fixed and stained.

To carry out KAT2A (GCN5) and/or KAT2B (PCAF) knockdown, Lipofectamine 2000 was used following the supplier's instructions. The siRNAs used were ON-Targetplus SMARTpools from Dharmacon (Thermo Fischer Scientific): GCN5L2 (KAT2A), L-009722-00; PCAF (KAT2B), L-005055-00; and non-targeting pool, D001810-10-05, as negative control. A unit of 60 nM siRNA was used in all knockdown conditions. Cells were treated with siRNAs for 72 h before collection.

Centrinone (PLK4 inhibitor) treatments were performed as described in ref. 62. Twenty-four hours after siRNA transfection, cells were treated for 24 h with centrinone at a final concentration of 125 nM.

### Immunofluorescence and microscopy for human cells

U2OS cells grown on glass coverslips were fixed for 7 min in −20 °C methanol, washed in PBS and blocked in 1% bovine serum albumin and 0.05% Tween-20 in PBS. Cells were incubated for 2 h at room temperature with primary antibodies, washed three times for 10 min in PBST (0.05% Tween-20 in PBS), incubated for 45 min at room temperature with secondary antibodies, stained with ∼1 μg ml^−1^ Hoechst 33258, washed three times in PBST and mounted. Primary antibodies are described above. Secondary antibodies were 1:1,000 goat anti-rabbit coupled to Alexa 488 and 1:1,000 goat anti-mouse coupled to Alexa 568. Imaging was done on a Zeiss LSM710 confocal microscope. Optical sections were acquired every 0.12 μm, and planes containing centrioles were projected together. Images were processed using ImageJ, preserving relative image intensities within a series.

Levels of PLK4-pS305 at the centrosome were quantified as follows. Cells were fixed with methanol, stained for PLK4-pS305 and γ-tubulin to localize centrosomes. The total PLK4-pS305 signal was determined within a circular region of 20 px diameter (∼1 μm) centred on the γ-tubulin focus. After subtraction of the background present outside cells, the total centrosomal intensity per cell was determined as the sum of all centrosomal intensities.

### Homology modelling

Crystallographic structures of PLK4 were taken from the Protein Data Bank (PDB accession codes: 3COK, 4JXF) for both the active and inactive forms of the protein^63^. Owing to a lack of electron density, no structure was resolved for residues in the activation loop, and so a structure was constructed for this segment using the Modeller programme^64^. In the active form, segments 158–189 and 212–226 were modelled using the structures of PLK2 and PLK3 as starting templates (accession codes: 4i6h and 4b6l, respectively). In the inactive form, residues 165–191 and 211–216 were modelled using an Aurora Kinase structure (accession code 4J8M) as a starting template. This latter structure was chosen because of its similarity with the fold of the N-terminal part of the activation loop of PLK4 (in the inactive structure of PLK4, see PDB ID 4JXF). In the 4JXF structure the DFG is in an inactive conformation^65^.

For each structure (active and inactive), 1,000 homology models were generated using the automodel module of Modeller^64^ and their quality was evaluated using the DOPE score^66^.

#### System set-up

The histidine protonation states were obtained using a protocol based on free energy perturbation calculations, as described in ref. 67. Except for histidines, all amino acids were built in their standard protonation states, as were the N- and C-termini of the kinase (NH3+ and COO−, respectively). Coordinates of ATP in the active form were obtained by replacing nitrogen by oxygen in the ANP ligand of structure 3cok. Hydrogen atoms were added using the Hbuild module^68^ of the CHARMM programme^69^. The system was then subjected to an energy minimization, and solvated in an 80 Å pre-equilibrated box of TIP3P water molecules. Neutralization was achieved by the random addition of one to eight chloride counter-ions.

#### Molecular dynamics

For each structure (active and inactive), we performed four replicate simulations of 50 ns of both the WT and the doubly acetylated form (K45acK46ac). In addition, we performed four replicate simulations of 50 ns for the active and inactive (K45RK46R) mutants. The total cumulated simulation time for the ensemble of the study is thus 1.2 μs. The simulations were performed at a temperature of 298.15 K in the NPT ensemble and were run with the NAMD programme^70^ using the CHARMM36 force field^71^. For the acetylated lysines, parameters from ref. 72 were used. Non-bonded interactions were truncated at a cutoff of 14 Å, using a switch function for van der Waals, and a shift function for electrostatics. Long-range electrostatic interactions were evaluated using the particle mesh Ewald algorithm. We used a 2fs integration step. The SHAKE algorithm was used to constrain bonds with hydrogen atoms.

#### Analysis

To assess the degree of conformational change undergone by the proteins in the molecular dynamics simulations, we calculated the root mean squared difference in conformation. The root mean squared difference plots show that the trajectories stabilized towards the 20 ns mark of the simulations, so all analyses were carried out on the 20–50 ns window. We calculated the percentage of existence (occupancy) of the internal H-bonds of the kinase, as well as those mediated by the ATP in the active form using the COOR HBOND module of the CHARMM programme^69^. Pairwise distances between the backbone and the side chain of each residue were calculated by using the distance between the Cα atom and the centre of mass of the respective side chain. This analysis was performed to detect subtle and local modifications of conformation. The analysis protocol relies on the detection of reproducible differences between two sets of simulations: WT versus variant. All differences mentioned in the text will thus correspond to reproducible differences, unless explicitly stated.

### Mass spectrometry analyses

For MudPIT analyses protein mixtures were denatured with urea, reduced, alkylated and digested with endoproteinase Lys-C (Roche), followed by modified trypsin digestion (Promega). Peptide mixtures were loaded onto a triphasic 100 μm diameter fused silica microcapillary column, packed with C18 reverse phase (Aqua, Phenomenex) and strong cation exchange (Partisphere SCX, Whatman) particles. Loaded microcapillary columns were placed in-line with a Quaternary Dionex Ultimate 3000 HPLC pump and a LTQ Velos linear ion trap mass spectrometer equipped with a nano-liquid chromatography (LC) electrospray ionization source (ThermoFischerScientific). A fully automated 12-step MudPIT run was performed as described^73^ during which each full MS scan (from 300 to 1,700 *m*/*z* range) was followed by 20 MS/MS events using data-dependent acquisition. Proteins were identified by database searching using SEQUEST^74^ within Proteome Discoverer 1.3. Tandem mass spectra were searched against a non-redundant protein sequence database for *Homo sapiens* containing 20,276 protein sequence entries (Uniprot release from 2010–09). Cysteine residues were considered to be fully carbamidomethylated (+57 Da statically added), methionine to be oxidized (+16 Da dynamically added) and lysine to be acetylated (+42 Da dynamically added). Three missed cleavages were permitted. Peptide mass tolerance was set at 2.5 and 0.8 Da on the precursor and fragment ions, respectively. Tandem mass spectra were filtered with XCorr values equal to 1.5 (+1), 2.5 (+2), 3.0 (+3) and 3.2 (>+3). Only peptides with a minimum length of seven residues and maximum deltaCn values of 0.3 were retained. Tryptic peptides containing C-terminal acetylated lysines were rejected, leading to a false discovery rate below 5%.

The LC/MS–MS analysis on the OrbiTrap ELITE were performed as follows: digested samples were analysed using an Ultimate 3000 nano-RSLC (Thermo Scientific, San Jose California) coupled in line with an LTQ-Orbitrap ELITE mass spectrometer via a nano-electrospray ionization source (Thermo Scientific, San Jose California). Peptide mixtures were loaded on a C18 Acclaim PepMap100 trap-column (75 μm ID × 2 cm, 3 μm, 100 Å, Thermo Fisher Scientific) for 3.5 min at 5 μl min^−1^ with 2% acetonitrile (ACN) and 0.1% formic acid (FA) in H_2_O, and then separated on a C18 Accucore nano-column (75 μm internal diameter (ID) × 50 cm, 2.6 μm, 150 Å, Thermo Fisher Scientific) with a 120 min linear gradient from 5 to 50% buffer B (A, 0.1% formic acid (FA) in H_2_O; B, 80% acetonitrile (acetonitrile (ACN)) and 0.08% FA in H_2_O) followed with 10 min at 99% B. The total duration was set to 150 min at a flow rate of 200 nl min^−1^. The temperature was kept constant at 40 °C. Peptides were analysed by Top 10-CID-HCD (collision induced dissociation and high-energy collisional dissociation) data-dependent MS. Tandem mass spectra were searched using SEQUEST HT within Proteome Discoverer 1.4, against a non-redundant protein sequence database for *H. sapiens* containing 27,858 protein sequence entries (Uniprot, release 2014–11). Cysteine residues were considered to be fully carbamidomethylated (+57 Da statically added), methionine considered to be oxidized (+16 Da dynamically added) and lysine considered to be acetylated (+42 Da dynamically added), and two missed cleavages were permitted. Peptide mass tolerance was set at 7 p.p.m. and 0.5(CID)/0.02(HCD) Da on the precursor and fragment ions, respectively. The minimum peptide length required was six residues. Proteins with at least two peptides were considered identified. The protein identification list was filtered at a false discovery rate below 1%.

To identify KAT2A/B-dependent acetylated proteins, candidates were considered as potential targets if they lost acetylation on KAT2A/B knockdown. To identify these protein targets, the pool of acetylated peptides identified after tandem MS analyses in five independent control samples (acetylome WT) was compared with the pool of acetylated peptides identified after tandem MS analysis in three independent replicates analyses of KAT2A/2B knocked down cells (acetylome KAT2 KD) Proteins were considered as potential KAT2A/B acetylated targets if they were absent in the three replicates acetylome KAT2 KD analyses and present in at least three of the five replicates of the WT acetylome.

Acetylation abundance factors were calculated as follows: PLK4 acetylated peptides specifically identified in KAT2A, KAT2B and KAT2A mutant conditions were extracted and compared. Spectral counts for similar acetylated peptides identified across these three conditions were divided by the total PLK4 spectral counts identified in each condition, to normalize for experimental variation encountered between independent runs. This calculation was performed for each acetylated peptide identified, and normalized values were then summed up to define the acetylation abundance factor of PLK4 in each condition tested.

### Bioinformatic analyses

The gene ontology term analysis of the 398 KAT2A/B targets was performed in Manteia^34^. Venn diagrams were generated using BioVenn http://www.cmbi.ru.nl/cdd/biovenn/. The sequence logo was generated with Weblogo 3.0 (http://weblogo.threeplusone.com/create.cgi). Sequence logos are a graphical representation of an amino-acid multiple sequence alignment. Each logo consists of stacks of symbols, one stack for each position in the sequence. In all, 2,501 distinct ‘Ks' from the list of 398 proteins were used in this analysis with the 10 aa upstream and 10 aa downstream. The K itself is not shown in these logos. A random selection of 5,243 sequences (K in the middle, 21 aa length, 0.05% of the total K) from the total human proteins was used for the comparison. The overall height of the stack indicates the sequence conservation at that position, while the height of symbols within the stack indicates the relative frequency of each amino or nucleic acid at that position. The PLK4 multiple protein sequence alignment was performed in ClustalW2 (http://www.ebi.ac.uk/Tools/msa/clustalw2). The output ALN file was further processed with BoxShade (http://www.ch.embnet.org/software/BOX_form.html).

### Data availability

The MS proteomics data have been deposited to the ProteomeXchange Consortium via the PRIDE^75^ partner repository with the dataset identifier PXD004669. The additional data that support the findings of this study are available from the corresponding authors on request.

## Additional information

**How to cite this article:** Fournier, M. *et al*. KAT2A/KAT2B-targeted acetylome reveals a role for PLK4 acetylation in preventing centrosome amplification. *Nat. Commun.* **7**, 13227 doi: 10.1038/ncomms13227 (2016).

**Publisher's note:** Springer Nature remains neutral with regard to jurisdictional claims in published maps and institutional affiliations.

## Supplementary Material

Supplementary Information Supplementary Figures 1-8

Supplementary Data 1 PKAT2A/KAT2B-dependent acetylome data
