# Supplementary material for: KAT2A/KAT2B-targeted acetylome reveals a role for PLK4 acetylation in preventing centrosome amplification
Source: Nat Commun. 2016 Oct 31;7:13227. doi: 10.1038/ncomms13227 (PMC5095585; doi:10.1038/ncomms13227)
Supplement: Supplementary Information — Supplementary Figures 1-8 [file ncomms13227-s1.pdf]

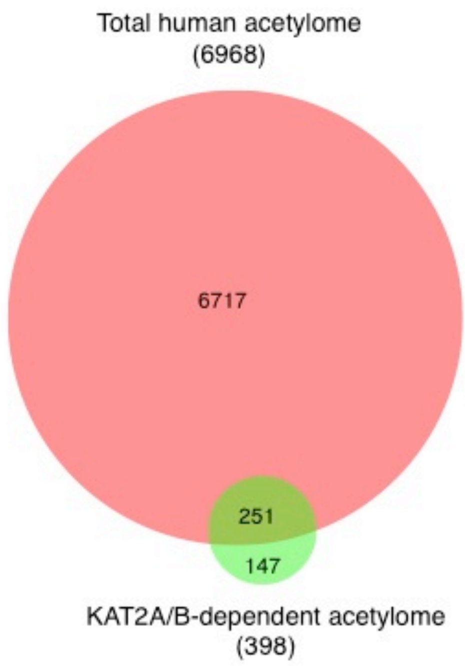

Supplementary Fig. 1. Comparative analysis of the global human acetylome with the KAT2A/2B-dependent acetylome. The total number of human acetylated proteins (6968) reported in PhosphositePlus[10], was compared with the 398 KAT2A/B-dependent acetylated proteins identified in our study. The overlap of the two datasets, represented by the intersection of the Venn diagram, shows that 63% of the KAT2/B-dependent acetylated proteins identified in our study were also found to be acetylated in other acetylome screens.

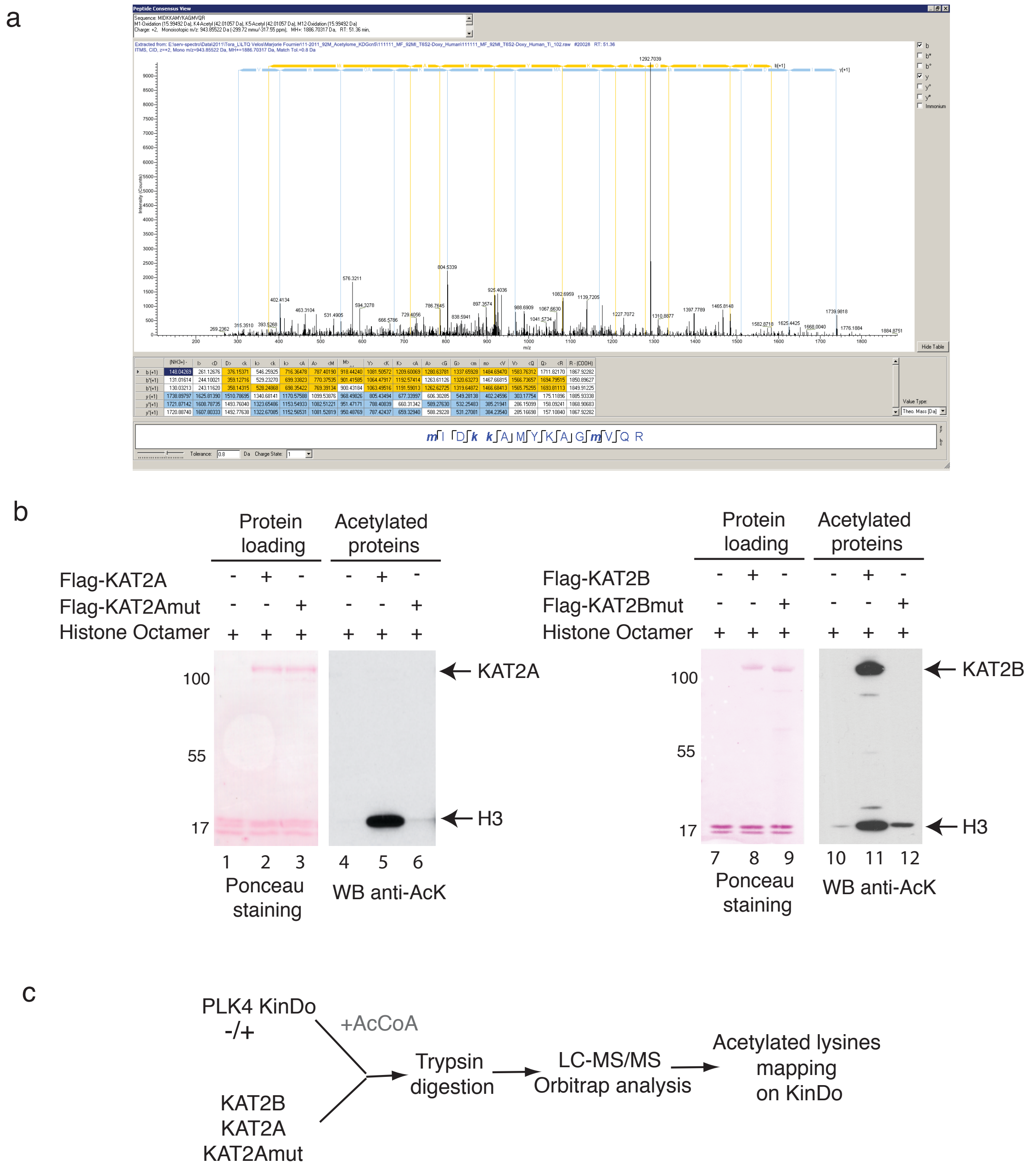

Supplementary Fig. 2. PLK4 kinase domain is acetylated by KAT2A/B at positions K45 and K46. **a**) Tandem mass spectrum of the PLK4 peptide acetylated at positions K45 and K46. **b**) In vitro acetyltransferase assay using purified histone octamers. Flag-KAT2A, Flag-KAT2Amut, Flag-KAT2B and Flag-KAT2mut were overexpressed in insect cells, and purified. 50 ng of proteins were then mixed with 50-100 ng of purified histone octamers in the presence of cold acetyl-coA. Proteins were separated, blotted and stained with Ponceau red (lanes 1-3 and 7-9). Western blot analysis (WB) was carried out with pan acetyl antibodies (α-AcK) (lanes 4-6 and 10-12). Molecular weight markers are indicated on the left in kDa. **c**) Schematic of the pipeline to identify sites acetylated on the PLK4 KinDo after in vitro acetyltransferase assay in the presence of recombinant purified KAT2s. Purified PLK4 KinDo was mixed either with recombinant purified KAT2A, KAT2B or KAT2A catalytically dead mutant, in the presence of cold acetyl-CoA. The reactions were further digested in solution by trypsin and analysed by LC-MS/MS on an Orbitrap Elite instrument .

a

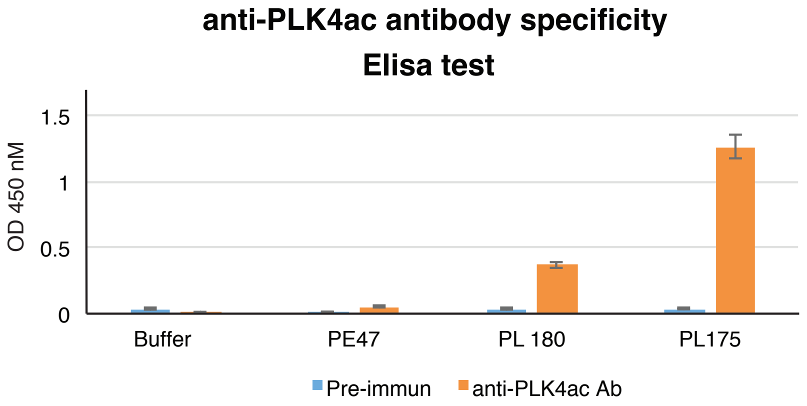

b

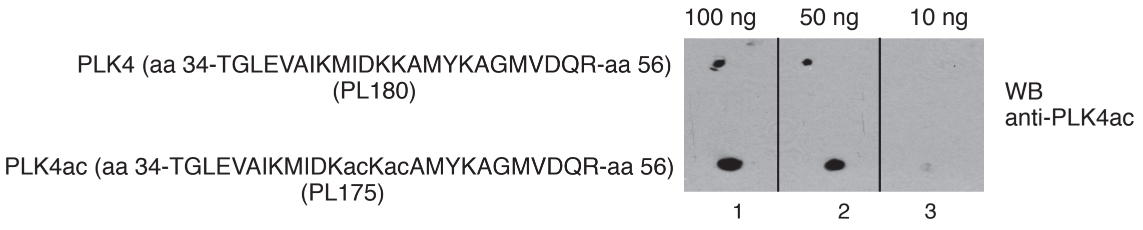

Supplementary Fig. 3 Characterization of the anti-PLK4ac antibodies.
a) The specificity of the purified rabbit polyclonal anti-PLK4ac antibody (see Materials) was tested by an ELISA test. The non-acetylated and the acetylated PLK4 peptides are depicted in panel b). The double acetylated histone H4 peptide PE47 (SGRGKacGGKacGLGKGGAKRHRKVLR) was used as a negative control. The anti-PLK4ac antibody was used in a 1/5000 dilution. Each test point was carried out in triplicates and the standard deviations were calculated.
b) The indicated amounts of PLK4 peptides, from aa 34 to aa 56 either non-acetylated (PL180, upper row), or acetylated (PL175, lower row), were deposited on a PVDF filter, dried and then revealed as a Western blot with the purified rabbit polyclonal anti-PLK4ac antibody (see Materials).

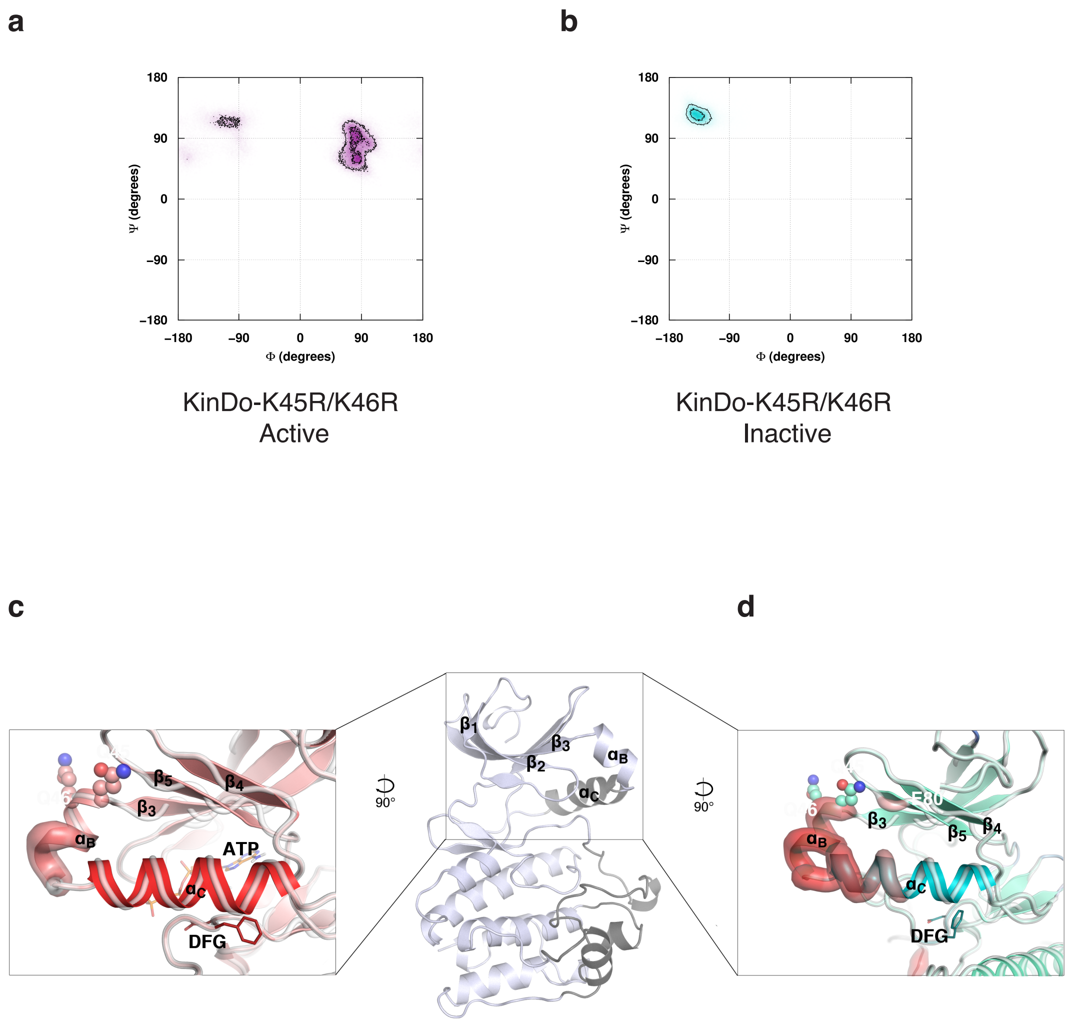

Supplementary Fig. 4. Structural dynamics simulations of the RR and the QQ mutants of the KinDo.

a) and **b**) Structural dynamics simulations of the K45R/K46R mutations of the KinDo show that the dynamics of the structure shifts to the inactive conformation. Ramachandran representation of the backbone dihedral angles of D154 (of the DFG motif, see also Fig. 5), in the WT and K45R/K46R mutated forms of the KinDo as analysed in the active (a) and inactive (b) conformations. See also Fig. 5 panels c-f.

c) and **d**) Structural dynamics simulations of the K45Q/K46Q46 mutations. The molecular dynamics simulations of the Q45Q46 mutant of PLK4 KinDo show local perturbations of the hydrogen-bond network around the mutation sites, both in the active (panel c) and in the inactive (panel d) conformations.

The region of increased fluctuations is represented in an enlarged ribbon drawing, highlighted in red. However, no long-range perturbations of these mutations were observed in the catalytic site, contrary to what was observed for the acetylated K45/K46 (see also Fig. 5).

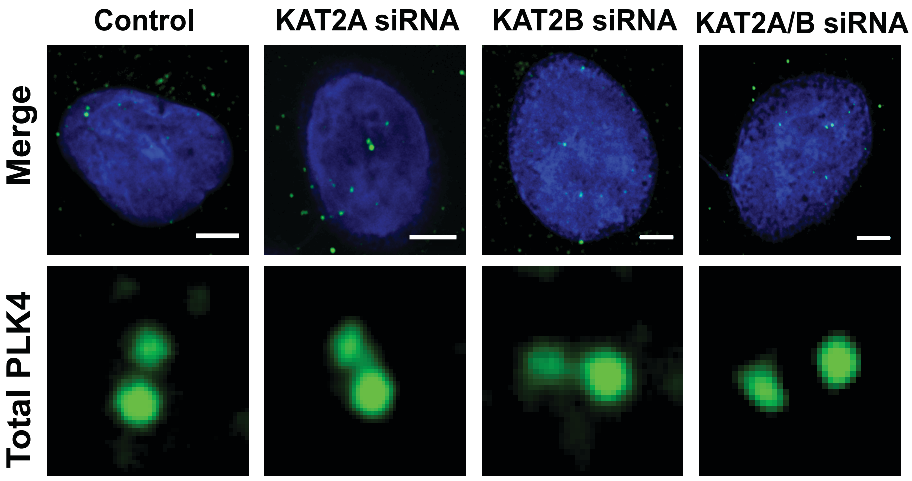

Supplementary Fig. 5. Analysis of centrosomal PLK4 upon KAT2A and KAT2B knock-down. Representative immunofluorescence analysis of PLK4 localization in control cells, or in cells in which KAT2A, KAT2B or both were depleted by using siRNA transfections.
Scale bars represent 5 µm.

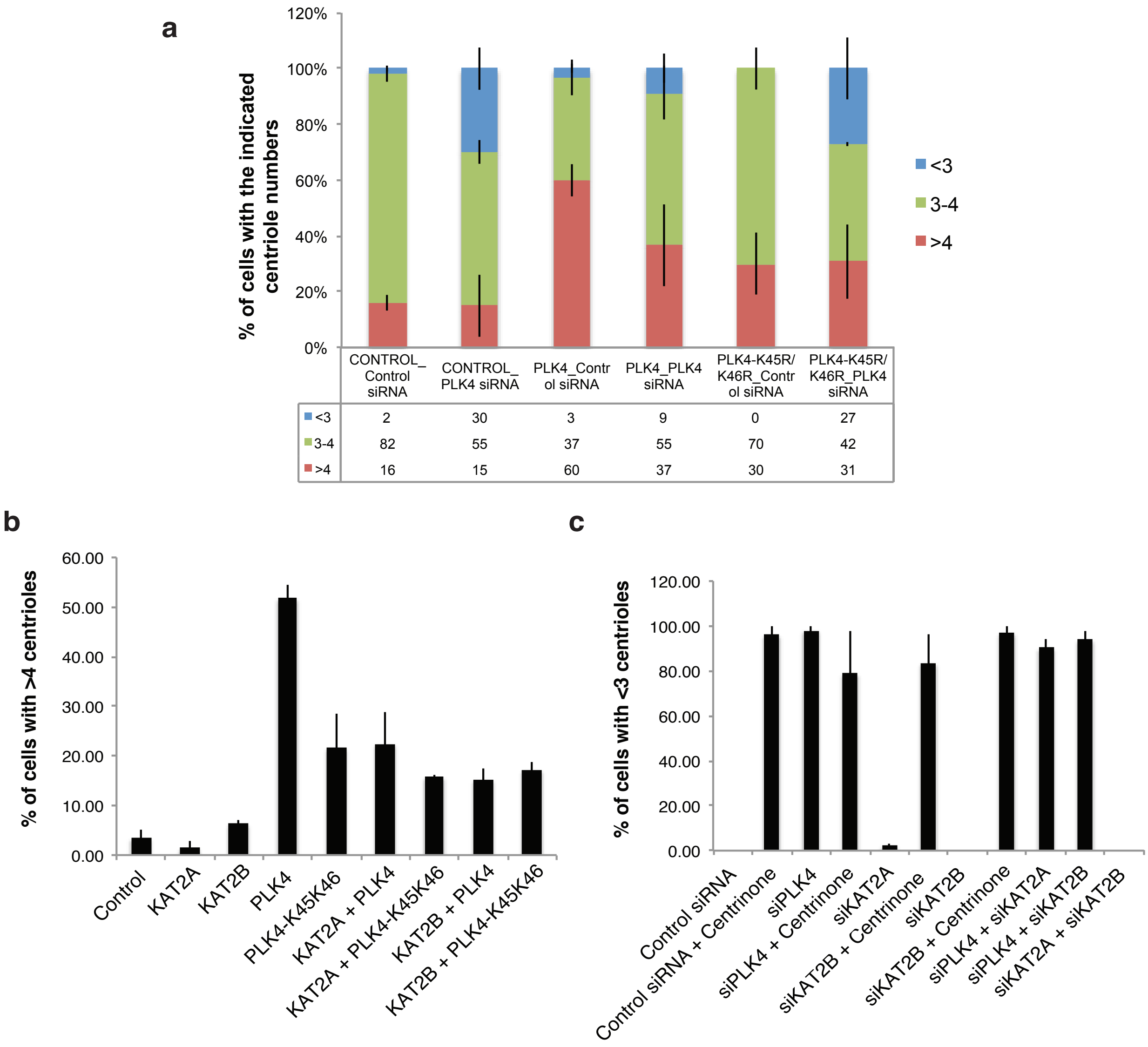

Supplementary Fig. 6. Centriole numbers upon depletion of ATAC complex components
a) U2OS cells were transfected with control siRNAs or siRNAs targeting the 3'UTR region of PLK4 (PLK4 3'UTR siRNA), to target endogenous PLK4. 24h after siRNA transfection, cells were transfected again with either a control-empty vector, a vector encoding full length PLK4-GFP, or full length PLK4-K45R/K46R-GFP, as indicated. Cells were fixed 48h later and stained for GFP (to visualize the exogenously expressed PLK4-GFP), and Centrin-2 (to visualize centrioles). The histograms report the average percentage of mitotic cells displaying >4 (red), 3-4 (green), or <3 (blue) centrioles.
The number of cells in each phenotypic category is given below the histogram. Data from >3 experiments, n>50 cells per condition. Error bars: standard error of the mean. **b**) U2OS cells were transfected with vectors overexpressing KAT2A, KAT2B, PLK4 or PLK4(K45K46R), alone or in combination, as indicated. 48h after transfection, cells were fixed and centrosomes visualized by immunofluorescence using antibodies against Centrin-2. The histograms report the average frequency of cells displaying aberrant Centrin-2 (>4) numbers. Data from >3 experiments, n>50 cells per condition. Error bars: standard error of the mean. Note that all cells were analyzed, without knowledge of their transfection status. **c)** U2OS cells were transfected with control siRNAs, siRNAs targeting the 3'UTR region of PLK4 (PLK4 3'UTR siRNA), or siRNAs targeting either KAT2A or KAT2B. 24h after siRNA transfection, cells were either left untreated or treated with the PLK4 inhibitor centrinone (125 nM)63. 48h after transfection (i.e. 24h after centrinone treatment), cells were fixed and stained for Centrin-2 to visualize centrioles. The histograms report the average percentage of mitotic cells displaying <3 centrioles. Data from >3 experiments, n>50 cells per condition. Error bars: standard error of the mean.

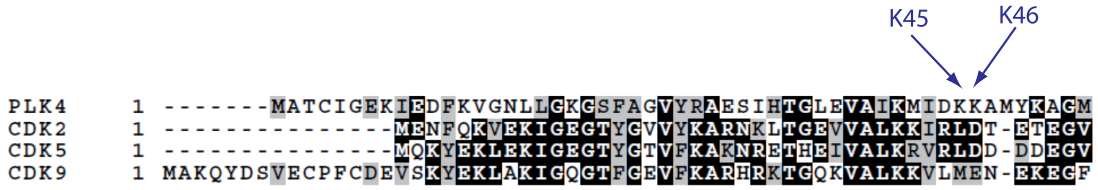

Supplementary Fig. 7. Protein sequence alignment of human CDK2, CDK5, CDK9 and PLK4 kinases. Acetylated lysine residues on the PLK4 sequence at positions K45 and K46, which are unique to PLK4, are indicated by arrows.

Supplementary Fig. 8

Fig1a

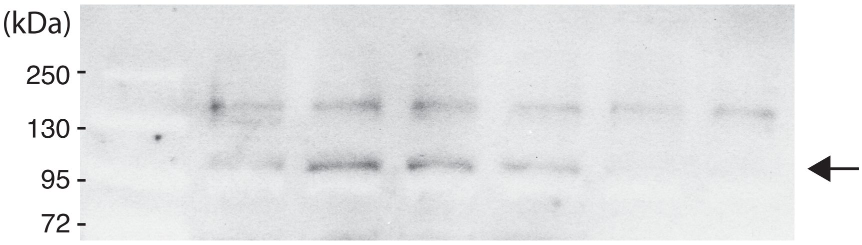

WB anti-KAT2A

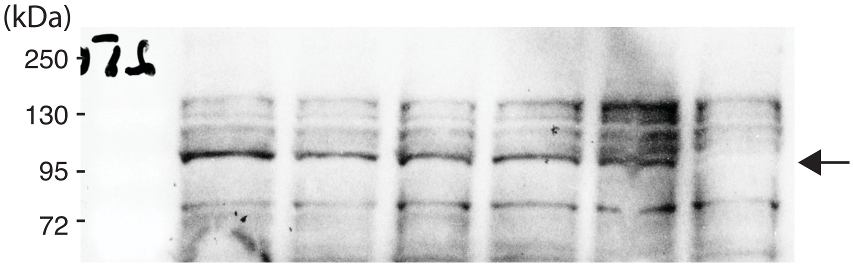

WB anti-KAT2B

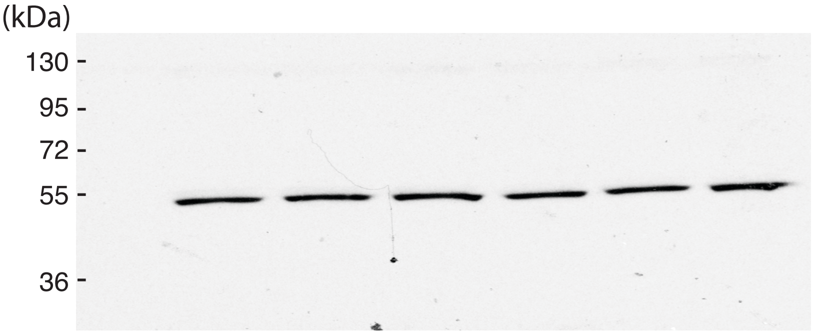

WB anti-γ-Tubulin

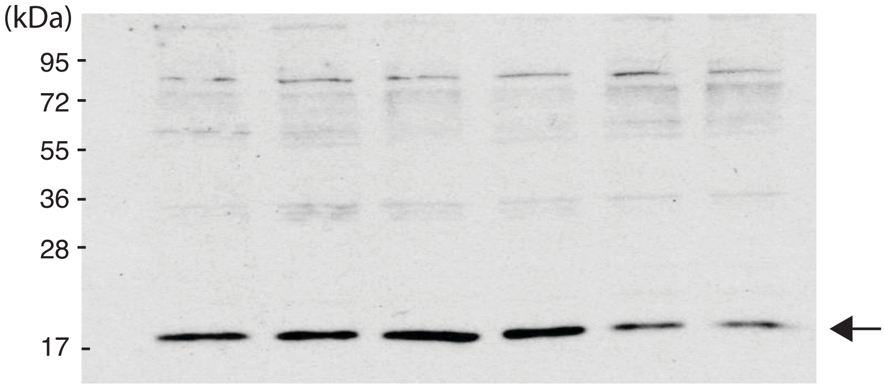

WB anti-H3K9ac

Uncropped panels Figure 1

Fig. 2d

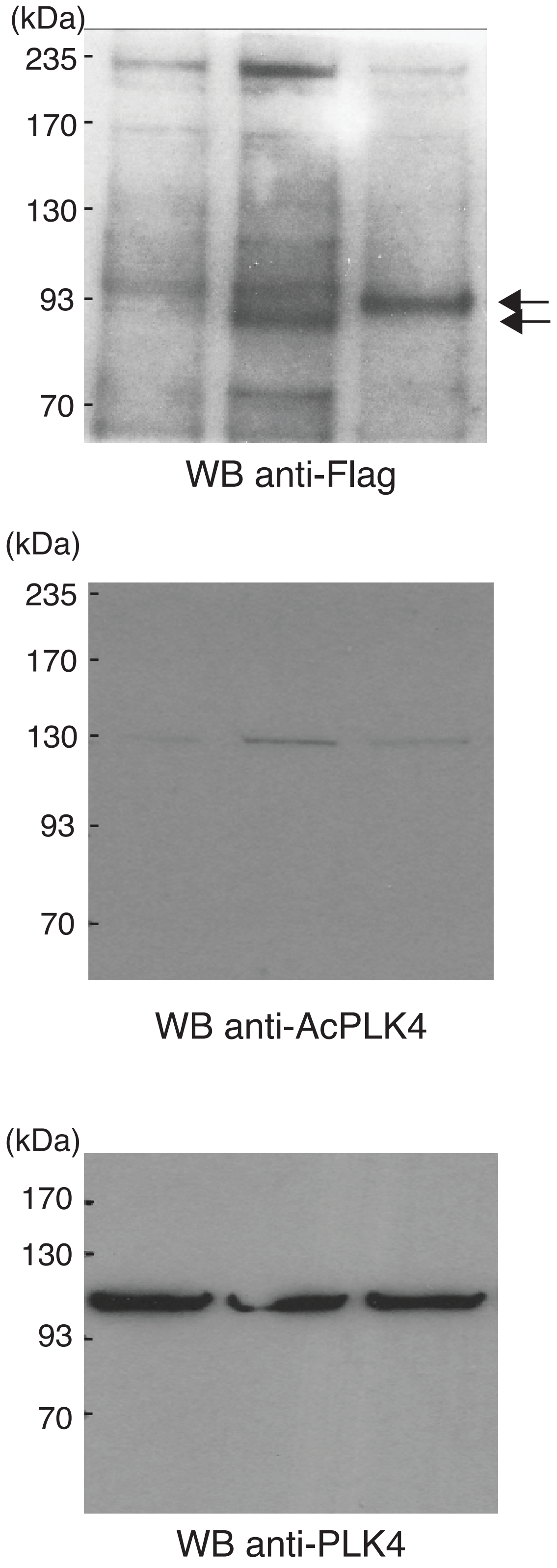

Uncropped panels Figure 2

Uncropped WB panels corresponding to Figure 3e

3e **CyclinE WB / WCL**

Time after double thymidine block (hr)

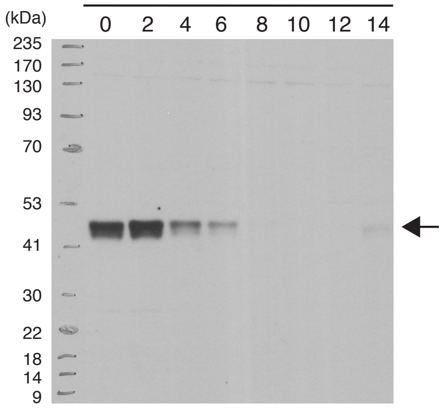

3e **Plk1 WB / WCL**

Time after double thymidine block (hr)

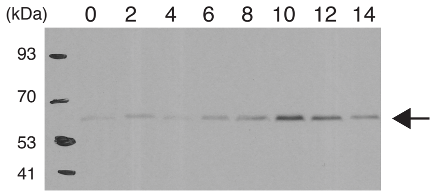

Uncropped WB panels corresponding to Figure 3f

3f **Kat2A WB/ Kat2A IP**

Time after double thymidine block (hr)

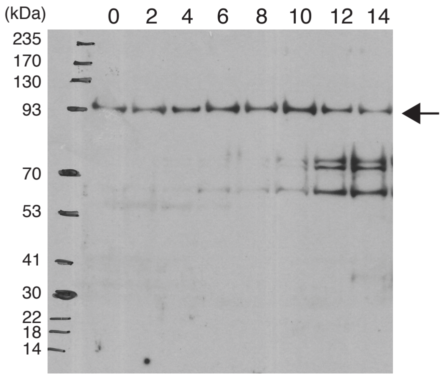

3f **Plk4 / Kat2A IP**

Time after double thymidine block (hr)

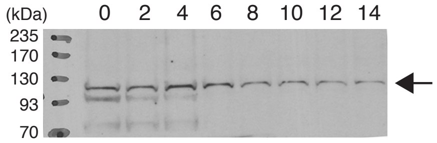

3f **Plk4 WB/ Mock IP**

Time after double thymidine block (hr)

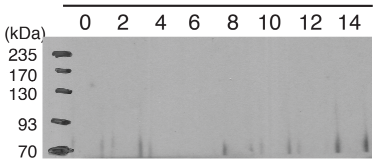

Uncropped panels corresponding to Figure 3

Fig. 6a

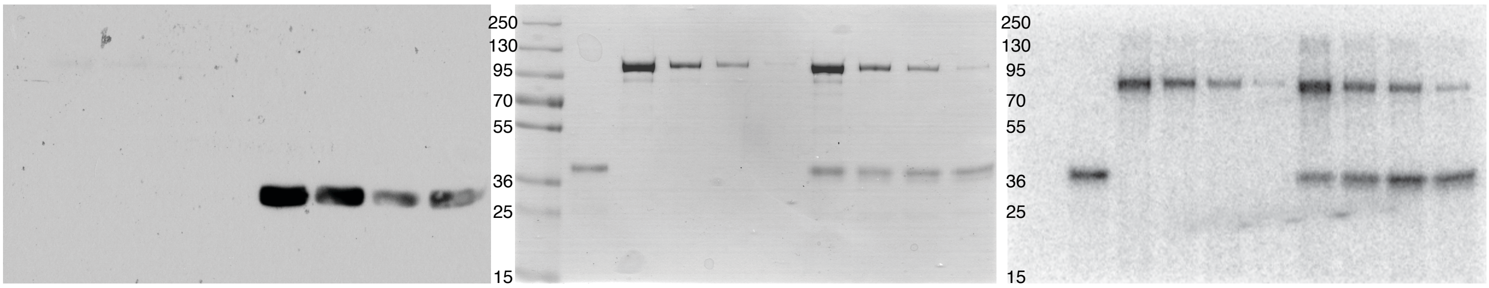

AcK-WB Coomassie blue staining ^{32}P

Fig. 6b

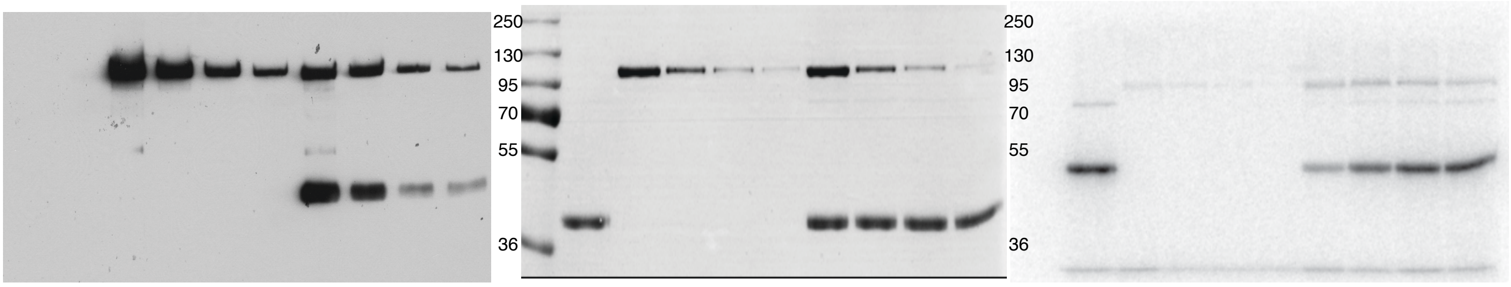

AcK-WB Coomassie blue staining ^{32}P

Fig. 6e

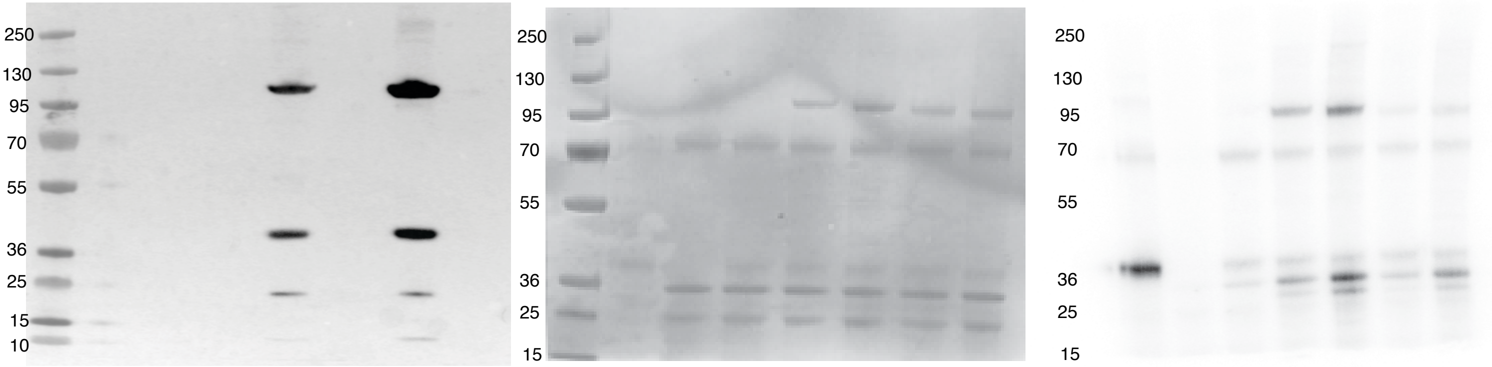

AcK-WB Coomassie blue staining ^{32}P

Fig. 6f

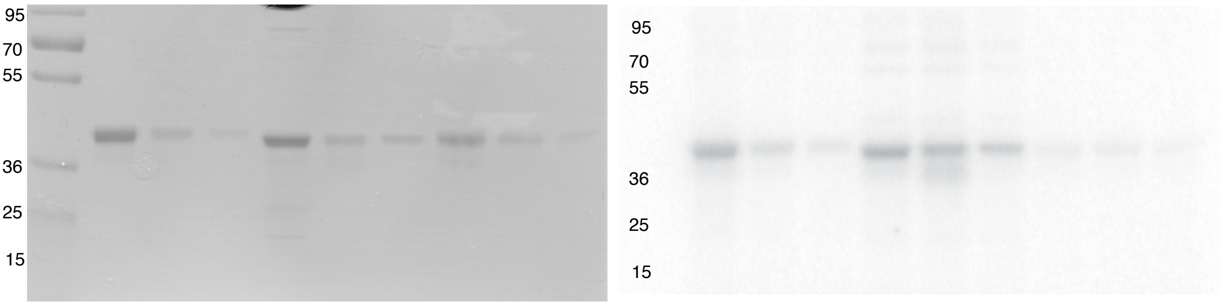

Coomassie blue staining ^{32}P

Uncropped panels Figure 6
